# Silk Fibroin Nanoparticles for Enhanced Cuproptosis and Immunotherapy in Pancreatic Cancer Treatment

**DOI:** 10.1002/advs.202417676

**Published:** 2025-03-17

**Authors:** Si Gao, Haodong Ge, Lili Gao, Ying Gao, Shuibin Tang, Yiming Li, Zhiqing Yuan, Wei Chen

**Affiliations:** ^1^ Department of Biliary‐pancreatic Surgery Renji Hospital Shanghai Jiaotong University School of Medicine Shanghai 200127 China; ^2^ Department of General Surgery Ruijin Hospital Shanghai Jiao Tong University School of Medicine Shanghai 200020 China; ^3^ Department of Pathology Xinhua Hospital Affiliated to Medicine School of Shanghai Jiaotong University Shanghai 200092 China; ^4^ School of Stomatology Inner Mongolia Medical University Hohhot Inner Mongolia Autonomous Region 010030 China; ^5^ Department of Medicine Mays Cancer Center University of Texas Health Science Center at San Antonio San Antonio TX 78229 USA

**Keywords:** cancer therapy, cuproptosis, elesclomol, silk fibroin, αPDL‐1

## Abstract

Cuproptosis is a newly discovered copper ion‐dependent programmed cell death. Elesclomol (ES) is a Cu^2+^ transporter that delivers Cu^2+^ into tumor cells, causing cell death at toxic doses. However, ES has a short blood half‐life, limiting its accumulation in tumors. This study introduces Tussah silk fibroin nanoparticles (TSF@ES‐Cu NPs) to protect ES and Cu^2+^. TSF, with a stable structure, resists metabolism in circulation. Targeting tumors with natural RGD peptides and TSF's unique secondary structure, enhances drug enrichment and special release in pancreatic tumors, improving treatment efficacy. In vitro, TSF@ES‐Cu induces tumor cell cuproptosis, releases DAMPs, promotes dendritic cells (DCs) maturation, and macrophage M1 polarization. In vivo, TSF@ES‐Cu reshapes the tumor microenvironment (TME), increasing mature DCs from 22.7% to 43.3%, CD8^+^ T cells from 5.08% to 17.1%, and reducing M2 macrophages from 50.7% to 18.4%. Additionally, the combined anti‐tumor efficacy of TSF@ES‐Cu and αPDL‐1 is 1.6 times higher than TSF@ES‐Cu alone and 2.5 times higher than αPDL‐1 alone. In summary, this study reports that the combination of TSF@ES‐Cu and αPDL‐1 effectively induces cuproptosis and reshapes the TME, offering a new approach for copper nanomaterial‐based tumor immunotherapy.

## Graphical abstract

Cuproptosis can be triggered via intracellular copper overload mediated by elesclomol (ES).Enhancing ES‐Cu's tumor targeting and circulation stability can efficiently induce tumor cuproptosis. TSF@ES‐Cu protect ES‐Cu, target tumors via natural RGD sequences, and release through multi‐responsiveness. TSF@ES‐Cu efficiently induce cuproptosis in tumor, promote antitumor immune cell infiltration in the tumor microenvironment, and enhance immunotherapy sensitivity when combined with immune checkpoint inhibitors.

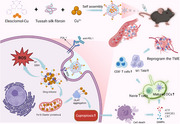

## 1 Introduction

Pancreatic Ductal Adenocarcinoma (PDAC) is a malignant epithelial digestive system tumor caused by non‐invasive precancerous lesions, and the five‐year survival rate for patients is below 9%.^[^ ^1^ ^]^ PDAC tissue comprises tumor cells and a complex tumor microenvironment (TME), which includes cellular components such as stromal cells and immune cells, as well as an extracellular matrix (ECM). It exhibits typical characteristics of excessive connective tissue proliferation and is an important pathological basis for PDAC's high invasiveness and drug resistance.^[^ ^2^ ^]^ PDAC cells have low immunogenicity and are not easily recognized by immune cells. In addition, due to the abundance of immunosuppressive cells, such as M2 macrophages, in the TME of pancreatic cancer, as well as the fibrotic characteristics of pancreatic tumor tissue and the presence of a stromal barrier that further limits immune cell infiltration, these factors collectively pose significant challenges for both traditional and novel immunotherapies in the treatment of pancreatic cancer.^[^ ^3^ ^]^ Traditional first‐line chemotherapy drugs such as gemcitabine often suffer from poor blood stability, low cell uptake rate, complex intracellular metabolism, and severe drug resistance, resulting in unsatisfactory clinical efficacy.^[^ ^4^ ^]^ At present, it is urgent to improve the efficacy of immunotherapy for pancreatic cancer and develop alternative treatment methods to improve the survival rate of pancreatic cancer patients.

Copper is a vital trace element crucial for cellular metabolism, but excess copper beyond the steady‐state threshold disrupts cellular function, leading to cell death.^[^ ^5^ ^]^ A novel copper‐dependent programmed cell death mechanism, “Cuproptosis,” occurs through intracellular copper ions (Cu^2^⁺) binding to lipidated mitochondrial proteins like dihydrolipoamide S‐acetyltransferase (DLAT) in the cellular tricarboxylic acid (TCA) cycle. This binding causes protein aggregation, downregulates Fe–S cluster proteins such as ferredoxin 1 (FDX1), induces protein toxic stress, and triggers programmed cell death.^[^ ^6^ ^]^ Cuproptosis also functions as immunogenic cell death (ICD), releasing damage‐associated molecular patterns (DAMPs) and tumor‐associated antigens that stimulate immune responses.^[^ ^7^ ^]^ This mechanism has the potential to reverse the immunosuppressive TME and enhance immune checkpoint inhibitors (ICIs), such as PD‐1 or αPD‐L1 therapies. Despite ICIs' success in melanoma, pancreatic ductal adenocarcinoma shows resistance due to immune escape and immunosuppressive TME.^[^ ^8^ ^]^ Studies reveal that intertumoral copper levels regulate PD‐L1 expression, and manipulating copper can improve αPD‐L1 therapy efficacy.^[^ ^9^ ^]^ Effective cuproptosis induction requires sustained Cu^2^⁺ accumulation in mitochondria, but this is tightly controlled by tumor cell mechanisms like glutathione and Cu^2^⁺ transporters, posing challenges.^[^ ^10^ ^]^ To address this, Cu ion carriers like disulfiram, 8‐hydroxyquinoline, pyridinethione, and elesclomol (ES) have been developed.^[^ ^11^ ^]^ ES selectively transports Cu^2^⁺ into cells, promotes mitochondrial accumulation, and induces apoptosis through ROS production.^[^ ^12^ ^]^ However, ES's therapeutic efficacy is limited by rapid bloodstream clearance, insufficient copper delivery to tumors, and inadequate toxicity.^[^ ^13^ ^]^ Extending ES's half‐life and enhancing targeted delivery could sustain Cu^2^⁺ accumulation, improving cuproptosis induction and anti‐tumor effects. Thus, increasing Cu^2^⁺ levels in tumor cells is a promising strategy for advancing cuproptosis‐based cancer therapies.

**Scheme 1:**
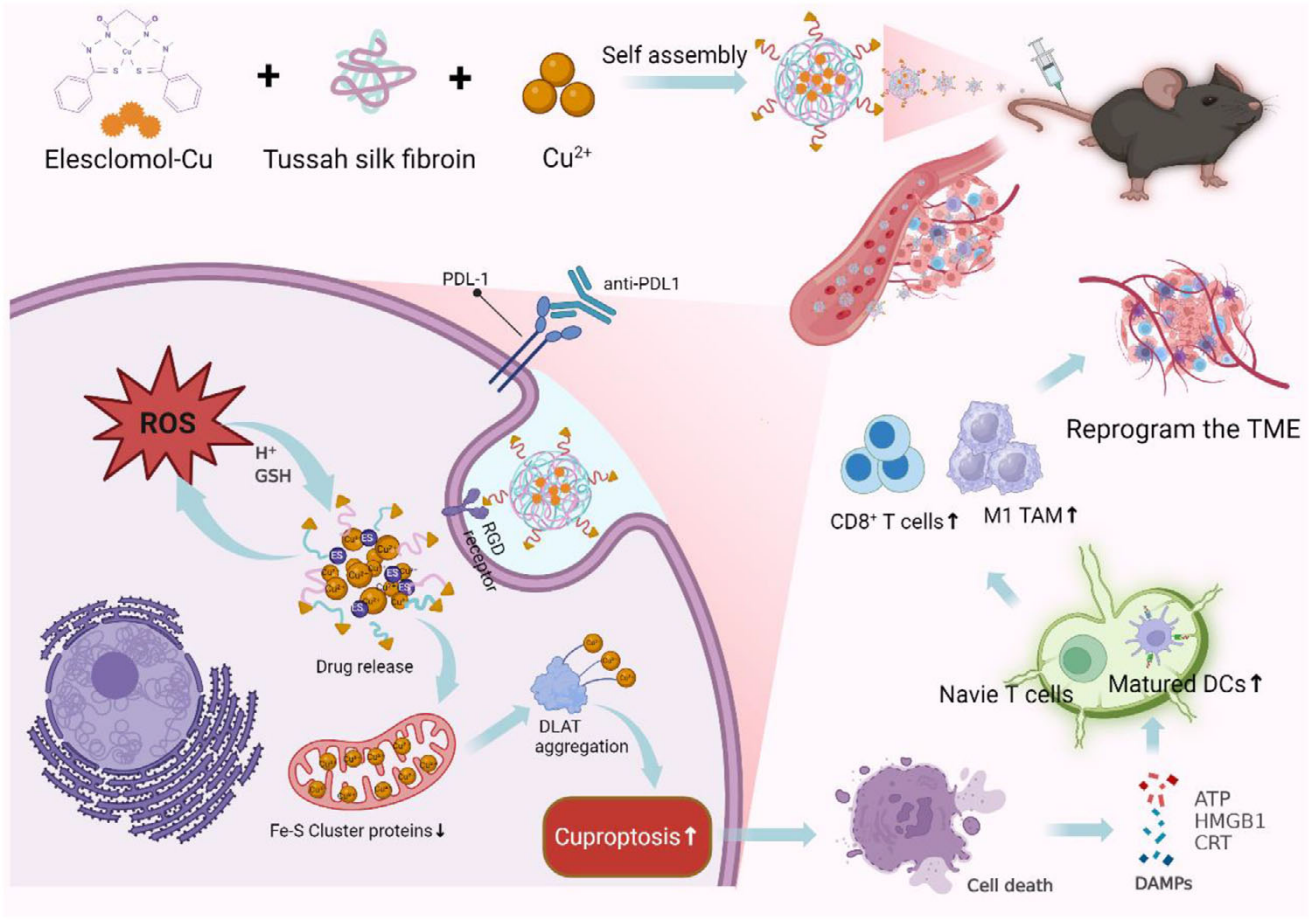
The synthesis of TSF@ES‐Cu NPs and its anti‐tumor mechanism (Created in https://BioRender.com).

Multi‐reactive nanomaterials have gained interest in cancer therapy, but their synthesis often involves toxic solvents and hazardous reactions, limiting medical applications.^[^ ^14^ ^]^ Tussah silk fibroin (TSF), a natural protein from Tussah silk, offers an alternative with excellent biocompatibility, non‐toxicity, and multi‐responsive properties.^[^ ^15^ ^]^ TSF nanoparticles (TSF NPs) leverage β‐folding and disulfide bonds to enable pH, GSH, and ROS‐responsive drug release in TME.^[^ ^16^ ^]^ Their surface features amino, carboxyl, and hydroxyl groups, allowing chemical modifications, and they contain arginine‐glycine‐aspartic acid (RGD) tripeptides, which target integrin receptors highly expressed on pancreatic cancer cells for enhanced specificity.^[^ ^17^ ^]^ Here, we developed TSF@ES‐Cu NPs by coordinating copper ions with TSF and encapsulating ES‐Cu complexes for controlled release in TME. The Cu^2+^ coordinated with TSF is also slowly released, elevating local Cu^2+^ levels and amplifying cuproptosis. Both in vitro and in vivo studies demonstrate that TSF@ES‐Cu NPs induce cuproptosis, triggering immunogenic cell death (ICD), promoting dendritic cell maturation, facilitating CD8^+^ T cell infiltration, and reducing M2 cells in tumor, which enhances anti‐tumor immune responses, overcomes tumor immunosuppression and remodels the tumor immune microenvironment. Combining TSF@ES‐Cu NPs with αPD‐L1 shows superior tumor‐killing effects, highlighting the promise of cuproptosis‐based strategies in advancing cancer therapy (**Scheme** **1**).

## 2 Results and Discussion

### 2.1 Synthetic and Characterization of the TSF@ES‐Cu NPs

**Figure 1:**
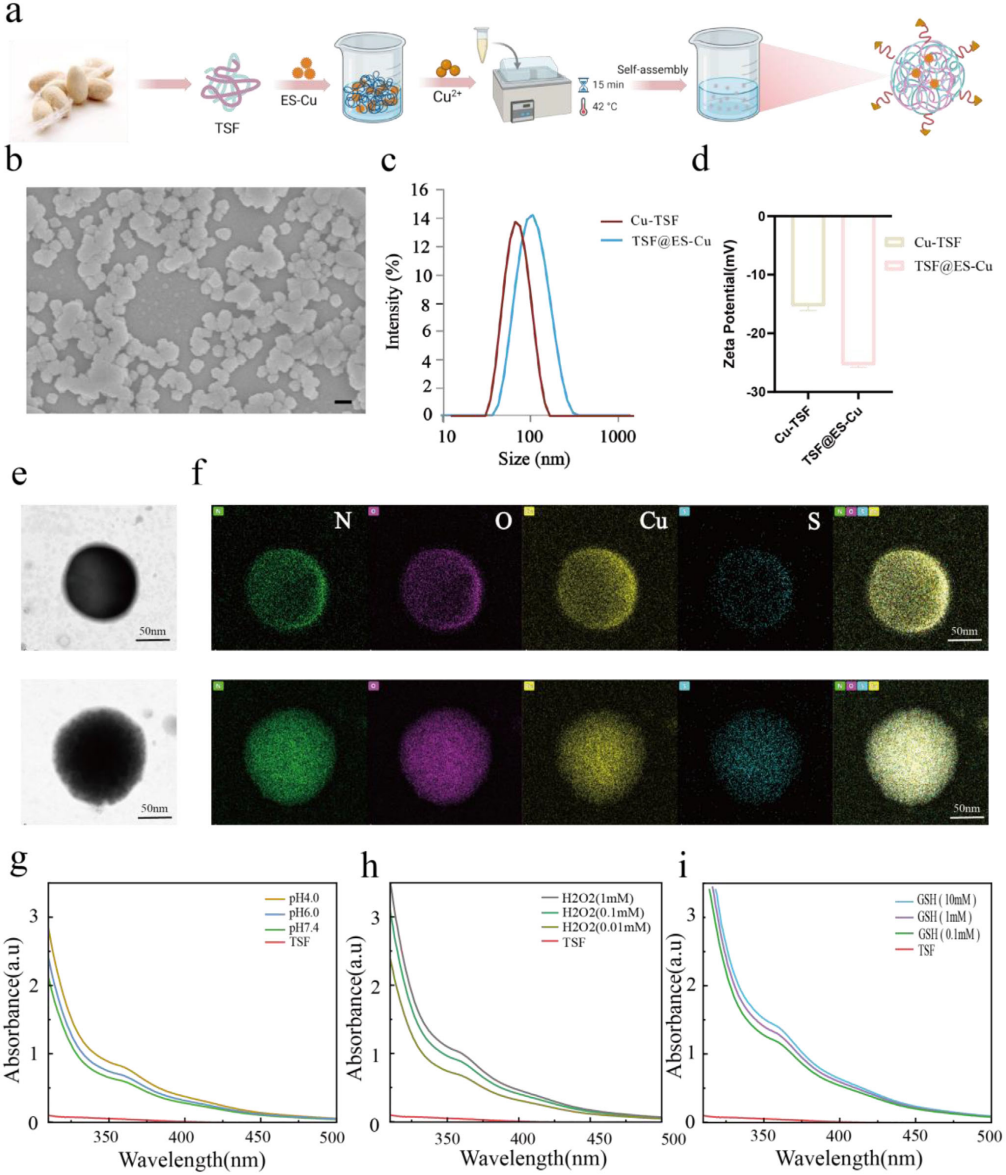
Synthetic and characterization of the TSF@ES‐Cu NPs. a) Illustrative representation of the synthesis process for TSF@ES‐Cu NPs (Created in https://BioRender.com). b) SEM of TSF@ES‐Cu NPs. Scale bar: 100 nm. The distributions of hydrodynamic particle sizes (c) and zeta potentials (d) for Cu‐TSF NPs and TSF@ES‐Cu NPs (measured in ultrapure water). e). TEM of Cu‐TSF NPs and TSF@ES‐Cu NPs. f) Representative element maps (Cu, O, N, and S) of Cu‐TSF NPs and TSF@ES‐Cu NPs. Scale bar: 50 nm. Alterations in absorption curves of ES‐Cu incubated with TSF@ES‐Cu at various pH(g), H_2_O_2_(h), and GSH (i), respectively.

Our preparation scheme for preparing TSF@ES‐Cu NPs is shown in **Figure** **1**a First, Tussah silk fibroin is extracted from natural tussah silk. Natural silk contains protein and sericin, which has immunogenicity; degumming treatment is required.^[^ ^18^ ^]^ According to previous research, we extracted silk fibroin protein and subjected it to degumming treatment. The degummed silk fibroin protein surface was relatively smooth, and then TSF NPs were quickly synthesized using a salt precipitation water bath method. Scanning electron microscopy (SEM) images show that Cu‐TSF NPs and TSF@ES‐Cu NPs formed into a uniformly sized spherical shape(Figure 1b), followed by the creation of an elemental composition diagram for display of TSF@ES‐Cu, compared to Cu‐TSF NPs, TSF@ES NPs contain not only Cu, O, N elements but also the unique S element of ES, confirming that Cu‐TSF NPs successfully encapsulate ES‐Cu(Figure 1e,f). Next, the size distribution of Cu‐TSF NPs and TSF@ES‐Cu NPs was assessed using the dynamic light scattering (DLS) technique. The mean hydrodynamic diameters of the nanoparticles are 80 nm (Cu‐TSF NPs) and 98 nm (TSF@ES‐Cu NPs), respectively (Figure 1c). The polydispersity index (PDI) is less than 0.3, suggesting that the nanoparticle size distribution is narrow and consistent. We have the zeta potentials of Cu‐TSF NPs and TSF@ES‐Cu NPs, ≈ −25 and −15 mV, respectively, indicating that they have electrostatic repulsion, keeping them stable in an aqueous solution (Figure 1d). After 35 days in a buffer solution with pH 6.8, the changes in hydrodynamic particle size and surface charge can be ignored, thus verifying this hypothesis (Figure S7, Supporting Information).

TSF is composed of a heavy (H) chain, a light (L) chain, and a P25 glycoprotein. The heavy and light chains are linked by a disulfide bond, while the hydrophobic domain of the H chain consists of repeated Gly‐X motifs, where X denotes the amino acids Ala, Ser, Thr, and Val. These sequences can form anti‐parallel β‐sheets, which play a key role in enhancing the stability and mechanical strength of the fibers.^[^ ^19^ ^]^ H^+^, H_2_O_2_, and GSH can reduce the β‐folding ability of TSF NPs, loosen their structure, and accelerate drug release.^[^ ^20^ ^]^ This is attributed to the direct disruption of hydrogen bonds in β‐ sheets and β‐crystals by H^+^ and H_2_O_2_, while GSH breaks the dauphine bonds within and between TSF molecular chains, promoting hydrogen bond damage and hydrolysis of β‐folding in aqueous solution. We know that the TME is a low pH and higher GSH‐containing environment than normal tissue. Therefore, TSF@ES‐Cu can release many drugs in tumors and increase the drug release rate with the production of ROS. We studied the drug release of TSF@ES‐Cu by simulating the TME. As shown in the drug release results, under acidic conditions and in the presence of H_2_O_2_ and GSH, we could detect more ES‐Cu release using a UV spectrophotometer, which confirms our previous discussion (Figure 1g–i).^[^ ^21^ ^]^ The above results demonstrate that we have successfully prepared TSF@ES‐Cu NPs with multiple responsiveness and the ability to encapsulate ES‐Cu.

### 2.2 The In Vitro Anti‐Tumor Performance and Cellular Uptake of TSF@ES‐Cu NPs

**Figure 2:**
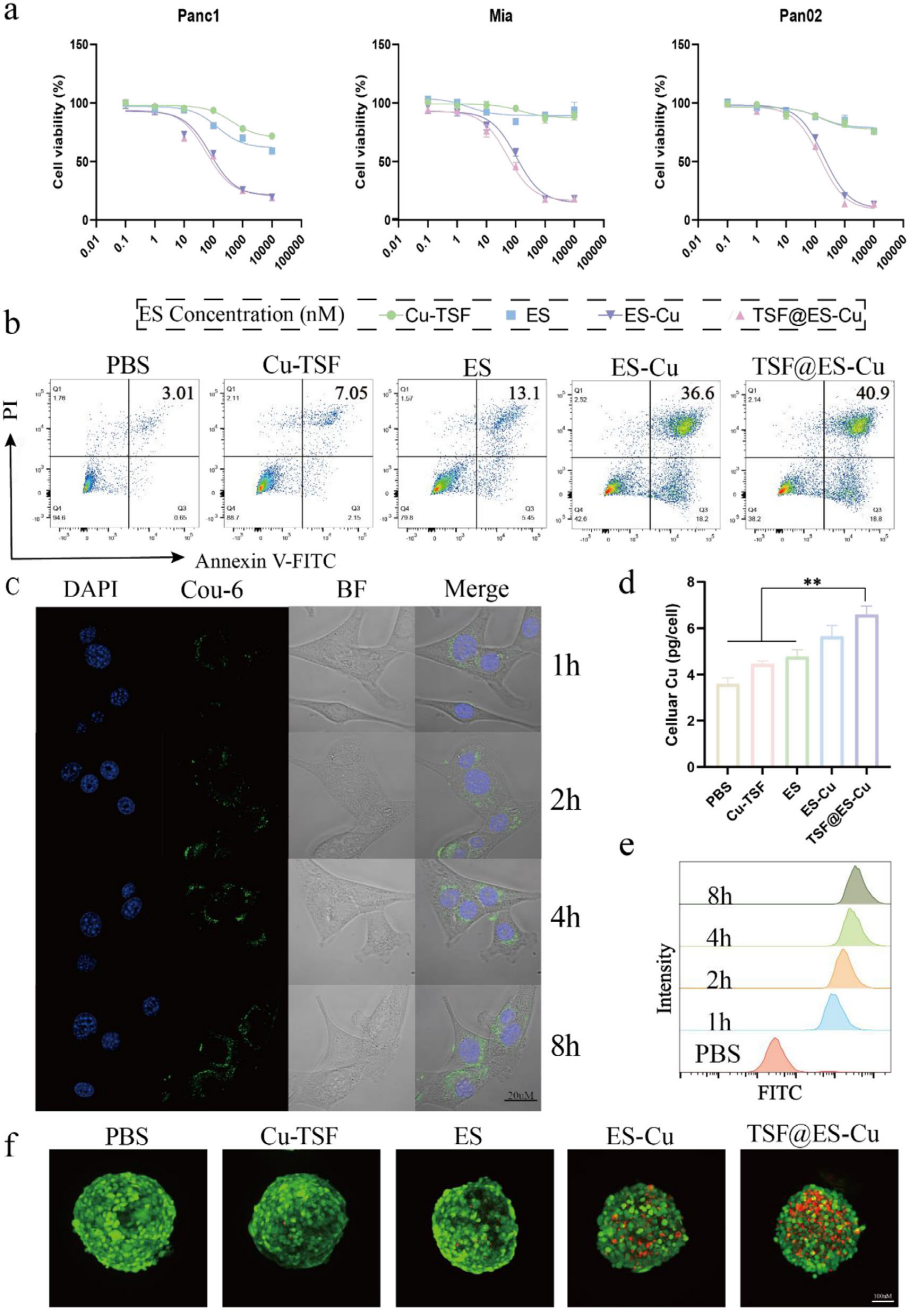
In vitro anti‐tumor activity and cellular uptake of TSF@ES‐Cu NPs. a) Cell viability of Pan02, Panc‐1, and Mia cells after various treatments. b) Representative FCM results illustrate the apoptosis rate of Pan02 cells after various treatments. c) Exemplary CLSM images of Pan02 cells co‐incubated with TSF@Cou‐6 for different durations (scale bars: 20µm). d) Intracelluar copper (Cu) uptake assay. e) FCM analysis of intracellular uptake of TSF@Cou‐6 NPs in Pan02 cells at 1, 2, 4, and 8 h. f) Exemplary CLSM images of 3D Pan02 cell spheroids treated with different formulations, stained with Calcein‐AM (green, indicating live cells) and PI (red, indicating dead cells), are shown.

First, we used the CCK8 assay to detect the anti‐tumor activity of different treatments on different pancreatic cancer cell lines (Mia, Panc‐1, Pan02). As shown in **Figure** **2**a, ES and Cu‐TSF NPs were almost non‐toxic to cells, but the IC50 value of ES‐Cu and TSF@ES‐Cu were all less than 200 nm. By calculation, we obtained the IC50 value of TSF@ES‐Cu in Mia, Panc‐1, and Pan02 cells were only 51.83, 57.45, and 67.76 nm, respectively. To further assess the cytotoxicity of TSF@ES‐Cu, the rate of apoptosis in Pan02 cells exposed to different treatments was evaluated using the Annexin V‐FITC/PI staining method. The data revealed that treatment with TSF@ES‐Cu induced a fivefold increase in apoptosis compared to the Cu‐TSF group and a threefold increase compared to the ES group (Figure 2b; Figure S10, Supporting Information). In addition, compared to traditional 2D monolayer cultures, 3D tumor spheroids offer a more accurate representation of in vivo treatment responses.^[^ ^22^ ^]^ Therefore, multicellular tumor spheroids were constructed to further examine the anti‐tumor effects of TSF@ES‐Cu using live/dead cell viability staining with Calcein‐AM and PI. As illustrated in Figure 2f, the ES and Cu‐TSF groups displayed intense green fluorescence (indicating live cells), while the ES‐Cu and TSF@ES‐Cu groups predominantly exhibited red fluorescence (indicating dead cells), with the TSF@ES‐Cu group showing the strongest red fluorescence. These observations align with the results of the previous cytotoxicity surveys.

The internalization of TSF@ES‐Cu nanoparticles was evaluated using both CLSM and FCM. TSF NPs were encapsulated with Cou‐6 (green fluorescence) and designated as TSF@Cou‐6, which was then co‐cultured with Pan02 cells for various durations. After one hour of incubation, significant green fluorescence signals were observed inside Pan02 cells by CLSM. Over time, the intensity of the green fluorescence increased, indicating a time‐dependent uptake of TSF@Cou‐6 NPs (Figure 2c). This was further confirmed by flow cytometry, which revealed that the fluorescence intensity at 8 h was 3.5 times higher than at 1 h and 1.2 times higher than at 4 h (Figure 2e). To investigate the correlation between Cu^2+^ accumulation and cytotoxicity, the copper content in Pan02 cells exposed to various formulations was quantified using a copper assay kit. The results indicated that the copper levels in cells exposed to TSF@ES‐Cu were 1.6 times greater compared to those in the PBS‐treated group. (Figure 2d). Overall, TSF@ES‐Cu NPs exhibited strong anti‐tumor activity, demonstrated excellent cellular uptake in both 2D cancer models and 3D tumor spheroid models, and effectively induced tumor cell death.

### 2.3 TSF@ES‐Cu can Effectively Induce Cuproptosis

**Figure 3:**
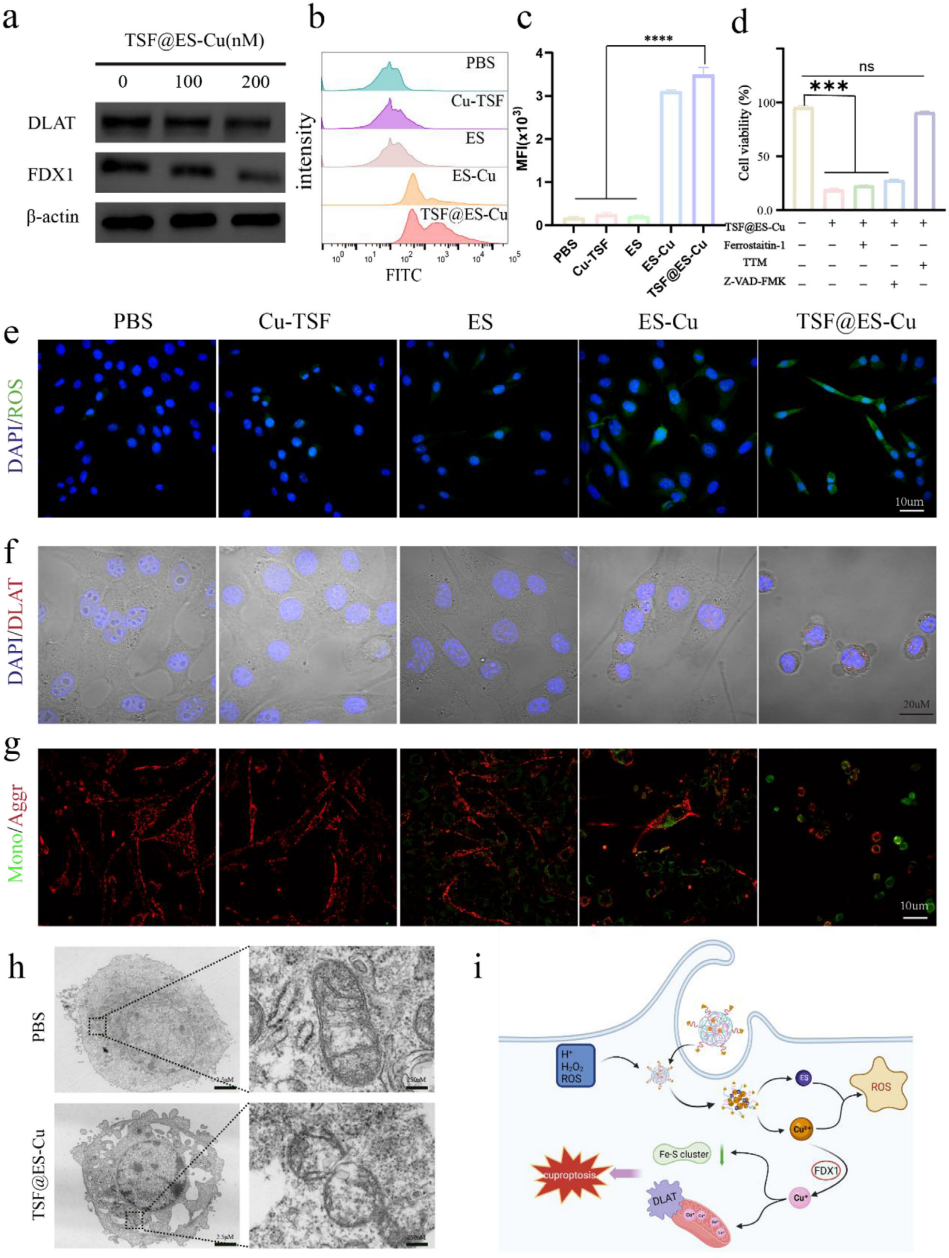
TSF@ES‐Cu effectively induces cuproptosis. a) Expression of DLAT and FDX1 detected by western blotting. b) Exemplary FCM patterns of ROS production in Pan02 cells with different treatments. c) Semi‐quantitative analysis of ROS levels. d) Cell viability of Pan02 cells after various treatments. e) Exemplary CLSM images showing ROS production in cells treated with various formulations. f) CLSM images showing the localization of DLAT protein in Pan02 cells through different treatments. g) Mitochondrial status was detected by JC‐1 staining. Scale: 30 um. h)Bio‐TEM images of Pan02 cells exposed to PBS or TSF@ES‐Cu NPs. Scale: 2.5 um. The mitochondria are indicated by the black box. Scale: 250 nm. i) Schematic diagram illustrating the proposed mechanism by which TSF@ES‐Cu induces cuproptosis (Created in https://BioRender.com).

Previous studies have shown that the reduced expression of FDX1 and the build‐up of DLAT are critical markers of cuproptosis, serving as defining characteristics of this particular type of cell death.^[^ ^6^ ^]^ A Western blot assay was performed to assess the expression levels of FDX1 and DLAT proteins in Pan02 cells treated with TSF@ES‐Cu. The results showed that FDX1 protein was downregulated, and DLAT protein was aggregated in Pan02 cells treated with TSF@ES‐Cu. This change became more pronounced with increased TSF@ES‐Cu concentration (**Figure** **3**a; Figure S15, Supporting Information). To further demonstrate the aggregation state of DLAT, we performed immunofluorescence analysis on Pan02 cells using CLSM and observed the changes in DLAT protein (red) after different treatments. We found that cells treated with PBS exhibited negligible DLAT accumulation, while those treated with TSF@ES‐Cu NPs showed significant DLAT accumulation (Figure 3f). Moreover, cell death induced by TSF@ES‐Cu treatment is not attenuated by other inhibitors but is specifically inhibited by copper‐induced cell death inhibitors (Figure 3d). Therefore, the research results indicate that TSF@ES‐Cu Could effectively induce cuproptosis.

Meanwhile, we evaluated the inhibitory effects of TSF@ES‐Cu NPs on tumor cell proliferation through colony formation assays and on tumor cell migration via scratch wound healing experiments. The results demonstrated that TSF@ES‐Cu NPs effectively suppressed both tumor cell proliferation and migration (Figures S12 and S13, Supporting Information). Furthermore, we examined the morphological changes in tumor cells treated with TSF@ES‐Cu at a concentration of 200 nm over different time intervals. By 6 h, the majority of tumor cells exhibited a morphological transformation from an elongated spindle‐like shape to a rounded configuration, accompanied by detachment from the culture surface. These observations indicate that TSF@ES‐Cu NPs possess a potent tumor‐killing effect (Figure S14, Supporting Information). Research has shown that ES and Cu^2+^ can induce tumor cells to produce ROS.^[^ ^12a^ ^]^ DCFH‐DA is a fluorescent probe that, upon oxidation by reactive oxygen species (ROS), is converted into DCFH, which subsequently emits green fluorescence. Therefore, we used DCFH‐DA to simultaneously detect ROS levels in Pan02 cells using CLSM and FCM after different treatments. As shown in the CLSM image, we found that TSF@ES‐Cu produced the greenest fluorescence (Figure 3e). Similarly, we concluded through FCM that TSF@ES‐Cu has the best ability to generate ROS, 16 times higher than PBS (Figure 3b,c).

Cuproptosis is accompanied by mitochondrial dysfunction. The characteristics of this process are a decrease in mitochondrial membrane potential (MMP), mitochondrial contraction, and a reduction or even loss of mitochondrial cristae.^[^ ^6^ ^]^ We use JC‐1 as a fluorescent indicator to detect changes in MMP. The red fluorescence indicates intact mitochondria with high mitochondrial membrane potential (MMP), whereas the green fluorescence represents dysfunctional mitochondria with reduced MMP. We found that TSF@ES‐Cu processed cells emit more green fluorescence (Figure 3g). Studies have shown that mitochondria in cells undergoing cuproptosis undergo morphological changes, indicating that cuproptosis can damage the mitochondria of cells.^[^ ^9b^ ^]^ To investigate the morphological alterations of mitochondria in Pan02 cells treated with TSF@ES‐Cu versus PBS, we employed a biological transmission electron microscope (TEM).TEM images showed that TSF@ES‐Cu could increase membrane density compared to PBS, leading to mitochondrial contraction, reduction, or even loss of mitochondrial cristae (Figure 3h). In summary, the above experiments have demonstrated that Pan02 cells successfully induced cuproptosis through TSF@ES‐Cu by regulating intracellular protein changes, upregulating intracellular ROS, and inducing mitochondrial damage.

### 2.4 TSF@ES‐Cu can Induce Immunogenic Cell Death in Tumors

**Figure 4:**
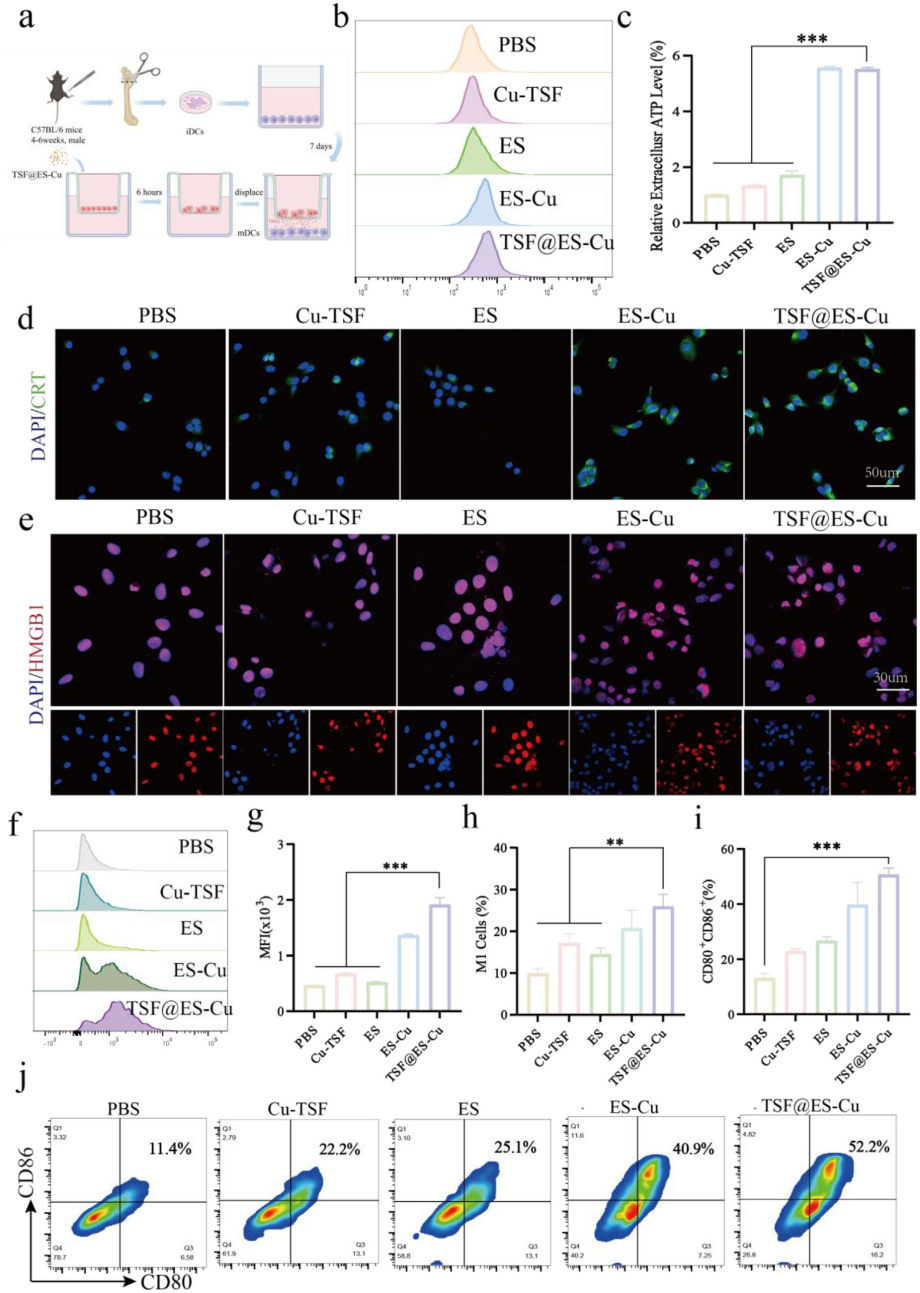
TSF@ES‐Cu Induces Immunogenic Cell Death in Tumor Cells. a) Schematic illustration showing the immunogenic cell death (ICD) effect induced by TSF@ES‐Cu NPs (Created in https://BioRender.com). b) Exemplary FCM profiles showing PD‐L1 expression in Pan02 cells following different treatments. c) Quantitative analysis of extracellular ATP levels in Pan02 cells exposed to different drugs. d) CLSM images showing the translocation of CRT to the cell membrane in Pan02 cells after different treatments (scale bar: 50 µm). e) CLSM images illustrating the nuclear localization of HMGB1 in Pan02 cells following different treatments (scale bar: 30 µm). f) FCM of CRT surface expression following different treatments. g) Quantification of CRT exposure levels as measured by FCM. h) Quantification of M1 macrophage in Pan02 cells following different treatments. i) Quantitative assessment of the BMDCs following different treatments. j) FCM analysis of the differentiation of BMDCs following various treatments.

Immunogenic cell death (ICD) represents a unique type of cell death that not only eliminates cells but also initiates a targeted immune reaction by releasing tumor‐associated antigens from the dying cells, thereby stimulating the host's immune system.^[^ ^23^ ^]^ Cuproptosis, a copper‐driven variant of immunogenic cell death (ICD), triggers immune activation by releasing tumor‐associated antigens and a range of damage‐associated molecular patterns (DAMPs), such as high‐mobility group box 1 (HMGB1), calreticulin (CRT), and adenosine triphosphate (ATP). These DAMPs can interact with antigen‐presenting cells, such as dendritic cells (DCs), facilitating antigen presentation to T cells and thereby promoting T‐cell‐mediated tumor cell destruction.^[^ ^24^ ^]^ Earlier research has demonstrated that cuproptosis can elevate the PD‐L1 expression on the membrane of cancer cells.^[^ ^9a^ ^]^ To further explore this, we evaluated PD‐L1 expression in Pan02 cells following treatment with different formulations using flow cytometry (FCM). The results showed a notable increase in PD‐L1 expression in cells treated with TSF@ES‐Cu compared to those treated with PBS (**Figure** **4**b; Figure S16, Supporting Information).

We studied a series of DAMPs produced by cuproptosis (Figure 4a). Compared to other groups, more exposed CRT (green fluorescence, known as the “eat me” signal) was detected on the cell membrane of cells treated by TSF@ES‐Cu (Figure 4d). We also detected the CRT exposure levels on the cell surfaces of different treatment groups using FCM (Figure 4f,g). Subsequently, we investigated the release of HMGB1 in the nucleus. As shown in CLSM images, the fluorescence intensity of HMGB‐1 (red fluorescence) inside the nucleus of Pan02 cells significantly decreased after TSF@ES‐Cu and ES‐Cu treatment. However, after treatment with PBS, Cu‐TSF, and ES, red fluorescence overlapped with stained DAPI nuclei (Figure 4e). In addition, we detected the extracellular ATP levels of Pan02 cells after different treatments using ATP detection kits. The results showed that the Pan02 cells treated with TSF@ES‐Cu showed the highest ATP content, five times higher than the PBS group (Figure 4c). The above experimental results indicate that TSF@ES‐Cu NPs can enhance cellular cuproptosis, which can release many DAMPs, demonstrating the enormous potential of TSF@ES‐Cu NPs to stimulate anti‐tumor immune responses.

Subsequently, Pan02 cells and immature dendritic cells (DCs) were also cultured in the transwell chambers. As shown in Figure 4j,i the proportion of mature DCs in the transwell chambers treated by TSF@ES‐Cu increased from 11.4% to 52.2% compared to the PBS group. The characteristic of TME is immunosuppressive properties, mainly due to the dominance of M2 anti‐inflammatory macrophages over M1 pro‐inflammatory macrophages in tumor‐associated macrophages (TAMs), which hinders the expansion and development of effector T cells.^[^ ^25^ ^]^ Therefore, promoting macrophage polarization from M2 to M1 is crucial for reversing immunosuppressive TME. To explore the potential immune properties of TSF@ES‐Cu NPs in vitro, Raw 264.7 cells were processed with interleukin‐4 (IL‐4) and polarized into M2 type TAMs, and then co‐culture with Pan02 cells treated with different methods by transwell technology. The results indicate that TSF@ES‐Cu can successfully polarize M2‐type TAMs into M1‐type TAMs, and the level of M1‐type TAMs has risen from 12.0% to 26.4% (Figure 4h). These findings support our hypothesis that TSF@ES‐Cu NPs can effectively trigger immunogenic cell death (ICD), leading to the release of damage‐associated molecular patterns (DAMPs). This, in turn, amplifies the immune profile of tumor cells and triggers anti‐tumor immune reactions.

### 2.5 TSF@ES‐Cu Biosafety and Biological Distribution

**Figure 5:**
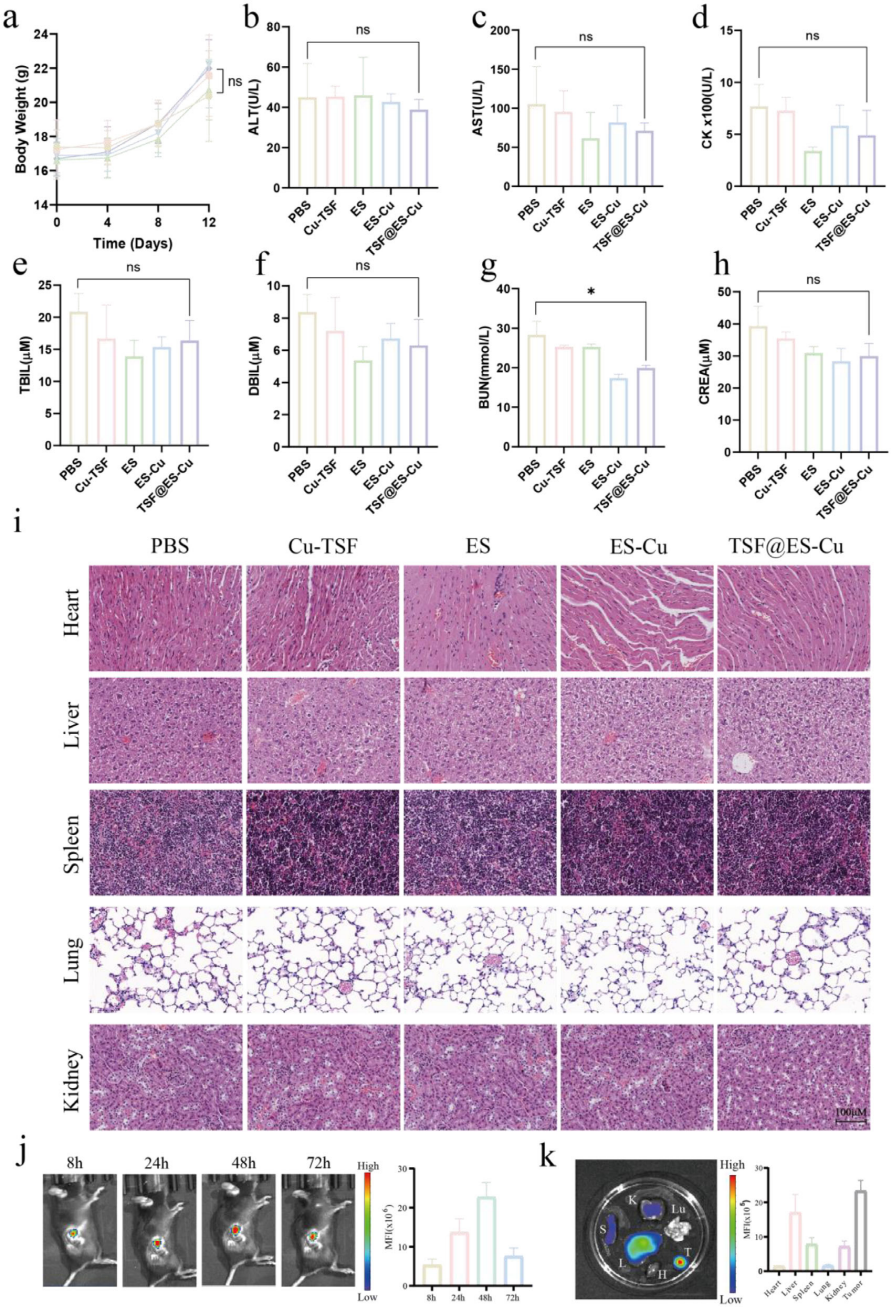
Biosafety and Distribution of TSF@ES‐Cu. a) Changes in body weight of C57/BL6 mice (n = 5) after treatment with PBS, Cu‐TSF, ES, ES‐Cu, or TSF@ES‐Cu. b–h) Biochemical assessment of blood plasma from mice administered various drugs (n = 3). b) ALT, c) AST, d) CK, e) TBIL, f) DBIL, g) BUN, and h) CREA. i) H&E staining assessment of the main organs from mice treated. j) Biodistribution of TSF@Cy5 NPs at various time points following intravenous injection. k) Ex vivo imaging of TSF@Cy5 in major organs and tumors (S, spleen; H, heart; Lu, lung; K, kidney; Li, liver; T, tumor).

We next assessed the biological safety and distribution of TSF@ES‐Cu in vivo. Mice were intravenously administered PBS, Cu‐TSF, ES, ES‐Cu, or TSF@ES‐Cu (at a total dose of 6 mg ES/kg) via tail vein injection, with four doses given consecutively. Before each injection, the mice were weighed. On day 15, blood and major organs were harvested from the treated mice for analysis. The findings showed that no substantial differences in body weight were observed across the various treatment groups, suggesting that TSF@ES‐Cu NPs exhibit minimal systemic toxicity (**Figure** **5**a). Further physiological and biochemical analyses were performed on the collected blood samples. The serum levels of various biomarkers, including ALT, AST, CK, TBIL, DBIL, BUN, and CREA, showed no significant differences between the TSF@ES‐Cu and PBS‐treated groups (Figure 5b–h). Remarkably, the BUN concentrations in the TSF@ES‐Cu group were significantly reduced compared to the PBS group (Figure 5g). Additionally, histopathological analysis using Hematoxylin and eosin (H&E) staining of vital organs showed no notable structural variations between the TSF@ES‐Cu and PBS‐treated groups (Figure 5i). These findings confirm that TSF@ES‐Cu NPs have low toxicity and demonstrate good biological safety.

Subsequently, we investigated the biological distribution of TSF NPs in the Pan02 tumor‐bearing mouse model. Encapsulate Cy5 in TSF NPs and name its TSF@Cy5, and the TSF@Cy5 nanoparticles were then administered into the tumor‐bearing murine model through intravenous injection via the tail vein, and fluorescence was detected using an imaging system. The results indicated strong fluorescence at 8 h post‐injection, with the highest intensity observed at 48 h. Even after 72 h, fluorescence aggregation still occurred, indicating that it exhibits a prolonged half‐life in vivo (Figure 5j). After 72 h, the mice were euthanized, and the fluorescence distribution of their main organs and tissues was observed. We can see that the fluorescence signal intensity in the tumor is the strongest, higher than that in the liver, kidney, heart, lungs, and spleen, indicating TSF@Cy5 has good performance in tumor targeting (Figure 5k). In summary, TSF NPs can effectively target and accumulate at the tumor site and have a long half‐life, which is highly advantageous for their anti‐tumor effects.

### 2.6 The Anti‐Tumor Efficacy In Vivo of TSF@ES‐Cu

**Figure 6:**
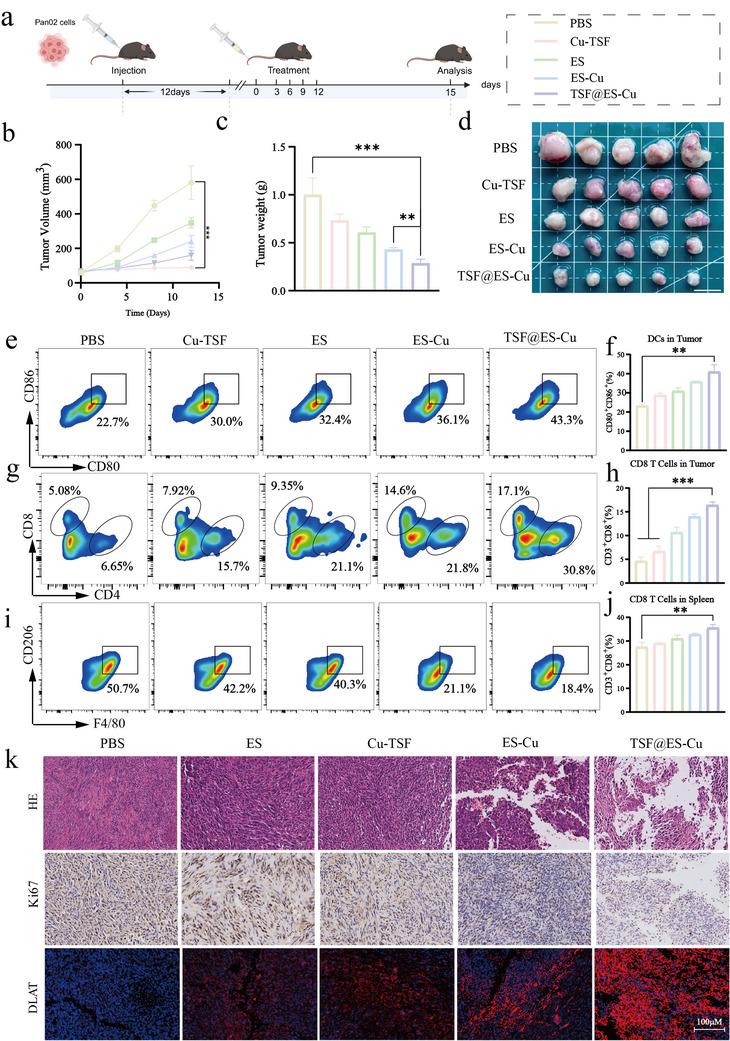
In vivo anti‐tumor efficacy of TSF@ES‐Cu. a) Schematic of the treatment schedule showing the intravenous administration of ES, ES‐Cu, and TSF@ES‐Cu (6 mg ES/kg) at specified time points, with n = 5 mice per group (Created in https://BioRender.com). b) Tumor growth was assessed through both tumor growth curves and c) ex vivo measurements of tumor weight. d) Representative images of tumors isolated from different treatment groups on day 15 are shown. Scale bar:1 cm. e) FCM of CD80^+^ and CD86^+^ dendritic cells (DCs), gated on CD11c^+^, within tumor samples. f) Quantification of CD80^+^CD86^+^ DCs in the tumors. g) FCM of CD8^+^ and CD4^+^ T cells, gated on CD3^+^, in tumor tissues. h) Quantitative assessment of T cell infiltration (CD8^+^) in tumor samples. i) FCM of M1 and M2 macrophages in tumor tissues under different treatment conditions. j) FCM of CD8^+^ T cells in spleen samples. k) H&E staining (upper panel), IHC staining for Ki67, and IF analysis of DLAT expression in tumor sections. Scale bar: 100 µm.

To investigate the efficacy of TSF@ES‐Cu in inhibiting pancreatic cancer, a subcutaneous tumor model was developed using Pan02 cells. Tumor‐bearing mice were randomly assigned to five different groups, each receiving four consecutive tail vein injections of either PBS, Cu‐TSF, ES, ES‐Cu, or TSF@ES‐Cu. The total dose of ES administered was 6 mg/kg. The tumor size and body mass were measured two days following each injection. The results indicated that TSF@ES‐Cu exhibited the most significant anti‐tumor effect, reducing tumor growth by a factor of four compared to PBS and by 1.5 times compared to ES‐Cu (**Figure** **6**b). On the third day after the end of treatment, the mice were euthanized, and their tumors were collected. Tumor images were captured, and the tumors were weighed. The mean tumor weight in the PBS‐treated group was 1 g, while the TSF@ES‐Cu group showed a significantly lower average tumor weight of 0.3 g, which was only 30% of that in the PBS group (Figure 6c,d). These findings indicate that TSF@ES‐Cu exhibits the highest anti‐tumor efficacy compared to the other treatments evaluated.

To investigate the immune response induced by TSF@ES‐Cu, we examined the tumors and spleens from mice in each treatment group. Initially, we assessed the maturation of tumor‐associated dendritic cells (DCs) by performing flow cytometry analysis. The findings indicated that the proportion of mature dendritic cells (CD80^+^CD86^+^) in the TSF@ES‐Cu‐treated group was 2.5 times higher compared to the PBS‐treated group, suggesting that TSF@ES‐Cu can significantly enhance DC maturation (Figure 6e,f). Since fully developed dendritic cells (DCs) play a vital role in antigen presentation and the activation of T lymphocytes, we next assessed T cell infiltration in the tumors. Flow cytometric analysis revealed that the proportion of CD3^+^CD8^+^ cytotoxic T cells in the TSF@ES‐Cu group was four times higher than that in the PBS‐treated group, further supporting the role of TSF@ES‐Cu in promoting immune activation within the TME (Figure 6g,h).Additionally, the analysis of CD8^+^ T cells in the spleen confirmed the results from the tumor tissue, with the TSF@ES‐Cu group showing a 1.2‐fold increase in CD8^+^ T cells compared to the TSF@ES‐Cu group (Figure 6j).

Tumor‐associated macrophages (TAMs) make up ≈50% of the tumor mass and are essential in driving tumor progression. They contribute to various processes, such as tumor growth, extracellular matrix remodeling, and immune suppression.^[^ ^26^ ^]^ TAMs are typically classified into two primary types: M1 macrophages, which are pro‐inflammatory, and M2 macrophages, which are anti‐inflammatory. M2‐TAMs are particularly abundant in tumor tissues and contribute to immune suppression by inhibiting T cell proliferation and facilitating tumor immune escape.^[^ ^27^ ^]^ To evaluate the effect of TSF@ES‐Cu on the polarization of TAMs in the TME, we analyzed the distribution of M1 and M2 macrophages in the treated tumors. M2 TAMs were identified as F4/80^+^CD206^+^, while M1 TAMs were represented by F4/80^+^CD206^−^. The findings demonstrated a marked decrease in the number of M2 macrophages in the TSF@ES‐Cu group, from 50.7% to 18.4%, in contrast to the PBS‐treated group, where M2 macrophages were 2.5 times more prevalent (Figure 6i; Figure S17, Supporting Information). These findings suggest that TSF@ES‐Cu, by inducing cuproptosis and immunogenic cell death (ICD), effectively reduces tumor cell proliferation and counteracts the immunosuppressive TME, thus enhancing the overall anti‐tumor response.

Subsequently, the therapeutic effect of TSF@ES‐Cu was confirmed by staining with H&E. Similarly, compared to other groups, The TSF@ES‐Cu group showed that the area of necrotic cells in tumor tissue was the largest (Figure 6k). In addition, quantitative analysis of Ki67 immunohistochemistry (IHC) staining confirmed the therapeutic effect of TSF@ES‐Cu. The expression level of Ki67 in the TSF@ES‐Cu was notably lower in comparison to the PBS group (Figure 6k). This means that the tumors in the TSF@ES‐Cu group have the worst cell proliferation ability. To evaluate the successful induction of copper deposition in vivo, we assessed the expression of DLAT in mouse tumor tissues through immunofluorescence staining stay. The strongest red fluorescence can be observed in the TSF@ES‐Cu group (Figure 6k). These results indicate that TSF@ES‐Cu can induce cuproptosis, triggering an immune response in vivo.

### 2.7 The Synergistic Enhancement of Anti‐Tumor Efficacy of TSF@ES‐Cu Combination with αPD‐L1

Immune checkpoint inhibitors such as αPDL1 can inhibit tumor cells by regulating the interaction between immune cells and tumor cells. Still, its effect is always limited by immunosuppressive TME, leading to its ineffectiveness in patients with pancreatic cancer.^[^ ^28^ ^]^ Nevertheless, we have confirmed that TSF@ES‐Cu not only reprograms tumor‐associated macrophages (TAMs) but also enhances the expression of PDL in cancer cells (Figure 4b). Therefore, we believe that TSF@ES‐Cu combined with αPDL‐1 has synergistic enhancement of anti‐tumor effect in vivo too. Subsequently, we established a subcutaneous model of Pan02 mice to validate the anti‐tumor effect of TSF@ES‐Cu combined with αPDL‐1.

**Figure 7:**
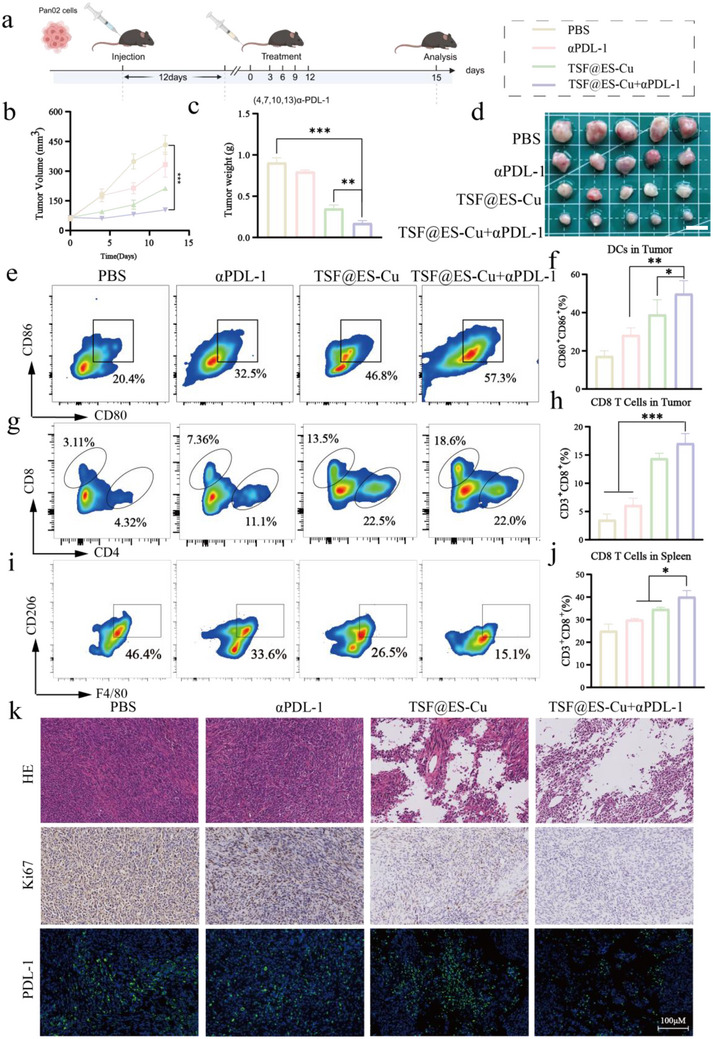
Synergistic Enhancement of Anti‐Tumor Efficacy of TSF@ES‐Cu Combined with αPD‐L1. a) Schematic representation of the treatment protocol, showing the timeline for intravenous injections and the biodistribution of TSF@ES‐Cu and TSF@ES‐Cu+αPDL‐1 (6 mg ES/kg for TSF@ES‐Cu, 10 mg αPDL‐1/kg, n = 5)(Created in https://BioRender.com). b) Tumor growth curves illustrate the progression of tumor volume across different treatment groups. c) Ex vivo tumor weight measurements after treatments. d) Representative images of isolated tumors from each group on day 15. Scale bar:1 cm. e) FCM of mature dendritic cells (DCs) in tumors, showing the gating strategy for CD80^+^ and CD86^+^ DCs. f) Quantification of CD80^+^ CD86^+^ DCs from the flow cytometry results in tumors. g) FCM analysis depicting the infiltration of CD8^+^ and CD4^+^ T cells, gated on CD3^+^ cells, within the tumor microenvironment. h) Quantitative analysis of CD8^+^ cell populations within tumors. i) FCM analysis of M1 and M2 macrophage phenotypes in tumor tissues from different treatment groups. j) FCM results show the distribution of CD8^+^ T cells in spleens. k) Representative H&E staining (top panel), along with immunohistochemical analysis of Ki67 expression and immunofluorescence detection of PD‐L1 in tumor tissues from various treatment groups. Scale bar: 100µm.

Mice with tumors were randomly assigned to four different treatment groups: PBS, αPDL‐1, TSF@ES‐Cu, and TSF@ES‐Cu + αPDL‐1. Each group received intravenous injections of the respective treatments for a total of four consecutive doses (6 mg ES/kg for TSF@ES‐Cu, 10 mg kg^−1^ for αPDL‐1). Tumor volumes and the body mass of the mice were recorded one day after treatments. The results demonstrated that the combination of TSF@ES‐Cu and αPDL‐1 exhibited the most pronounced anti‐tumor effect, achieving an anti‐tumor response 2.5 times greater than that of αPDL‐1 alone and 1.6 times greater than TSF@ES‐Cu (**Figure** **7**b,d). Upon completion of the treatment, the mice were sacrificed, and their tumors were excised and weighed. The mean tumor weight in the TSF@ES‐Cu + αPDL‐1 group was 0.17 g, which was only 50% of the tumor weight observed in the TSF@ES‐Cu group (Figure 7c). These findings suggest that the combination treatment of TSF@ES‐Cu with αPDL‐1 has superior anti‐tumor efficacy compared to TSF@ES‐Cu alone.

To further explore the immune response induced by these treatments, we analyzed tumors and spleens from the different groups. The findings showed that the proportion of mature dendritic cells (CD80^+^CD86^+^) in the TSF@ES‐Cu+αPDL‐1 group was significantly higher than in the groups treated with either αPDL‐1 or TSF@ES‐Cu alone, indicating that this combination treatment effectively enhances DC maturation (Figure 7e,f). Further analysis of tumor‐infiltrating T cells revealed that the proportion of CD3^+^CD8^+^ T cells in the TSF@ES‐Cu+αPDL‐1 group was 1.3 times greater than that in the TSF@ES‐Cu group (Figure 7g,h). Interestingly, the proportion of M2 macrophages was also found to be 1.2 times lower in the TSF@ES‐Cu+αPDL‐1 group compared to TSF@ES‐Cu alone (Figure 7i; Figure S18, Supporting Information). Additionally, the analysis of CD8^+^ T cells in the spleen confirmed the results from the tumor tissue, with the TSF@ES‐Cu+αPDL‐1 group showing a 1.5‐fold increase in CD8^+^ T cells compared to the TSF@ES‐Cu group (Figure 7j).

Subsequently, the therapeutic effect of TSF@ES‐Cu+αPDL‐1 was confirmed by staining with hematoxylin and eosin (H&E). Similarly, compared to other groups, the TSF@ES‐Cu+αPDL‐1 group showed that the area of necrotic cells in tumor tissue was the largest (Figure 7k). In addition, quantitative analysis of Ki67 immunohistochemistry (IHC) staining confirmed the therapeutic effect of TSF@ES‐Cu+aPDL‐1. We observed that the Ki67 expression in the TSF@ES‐Cu + αPDL‐1 group was notably lower compared to the PBS‐treated group (Figure 7k). At the same time, we evaluated the content of PDL‐1 on the tumor tissue surface of different treatment groups through IF. The results indicated that the green fluorescence in the TSF@ES‐Cu+αPDL‐1 group was notably reduced compared to the PBS group (Figure 7k). In summary, these findings suggest that TSF@ES‐Cu+αPDL‐1 can synergistically enhance anti‐tumor effects.

## 3 Conclusion

In this work, we developed multiple stimulus‐responsive tussah silk fibroin nanoparticles (TSF NPs), which can effectively deliver copper complexes to pancreatic cancer to induce cuproptosis of cancer cells. We synthesized tussah silk fibroin nanoparticles by extracting silk fibroin protein from natural tussah silk, which has the following advantages: First, it has good biocompatibility, biodegradability, and ease of processing. Second, due to its β‐folding region and disulfide bond that is easily disrupted by H^+^, GSH, and H_2_O_2_, TSF NPs can release drugs in specific tumor environments. Finally, it also contains rich RGD tripeptide, which can specifically target pancreatic cancer cells. We use TSF NPs encapsulated copper complex (ES‐Cu) to treat pancreatic cancer. TSF@ES‐Cu NPs transport copper ions into pancreatic cancer cells to produce rich ROS, destroy copper homeostasis of cells, down‐regulate FDX1 protein level, and induce DLAT protein aggregation, thus leading to cuproptosis of cells. Moreover, both in vitro and in vivo experiments have shown that TSF@ES‐Cu can induce the remodeling of the tumor immune microenvironment, including promoting the maturation of DCs, increasing tumor infiltration of CD8^+^T cells, and reducing the M2 ratio. TSF@ES‐Cu can increase the expression of PDL‐1 in pancreatic cancer cells; the combination of αPDL‐1 and TSF@ES‐Cu has a synergistic effect in treating tumors. In summary, this study reports that the combination of TSF@ES‐Cu and αPDL‐1 can effectively induce cuproptosis of pancreatic cancer cells and reshape the tumor immune microenvironment, providing a new direction for tumor immunotherapy based on copper nanomaterials.

## Conflict of Interest

The authors declare no conflict of interest.

## Supporting information



Supporting Information

## Data Availability

The data that support the findings of this study are available from the corresponding author upon reasonable request.
