# Supplementary material for: Silk Fibroin Nanoparticles for Enhanced Cuproptosis and Immunotherapy in Pancreatic Cancer Treatment
Source: Adv Sci (Weinh). 2025 Mar 17;12(18):2417676. doi: 10.1002/advs.202417676 (PMC12079484; doi:10.1002/advs.202417676)
Supplement: Supplementary file 1 — Supporting Information [file ADVS-12-2417676-s001.docx]

**Supporting information**

Si Gao^1^, Haodong Ge^2^, Lili Gao^3^, Ying Gao^4^, Shuibin Tang^1^, Yiming Li^1,5^, Zhiqing Yuan^1^, Wei Chen^1*^

**1.Materials and methods**

**Materials**

Tussah silk was purchased from Dandong, sodium carbonate (NaCO_3_), Lithium bromide(LiBr), cupric chloride(Cucl_2_), glutathione(GSH), and Hydrogen Peroxide (H_2_O_2_) were obtained from Shanghai Aladdin, Co. Dihydrolipoamide S-acetyltransferase (DLAT) polyclonal antibody, ferredoxin 1 (FDX1) anti-CRT, anti-HMGB1 were purchased from Cell Signaling Technology, ltd. Cell Counting kit-8, penicillin-streptomycin, fetal bovine serum (FBS), Dulbecco’s modified eagle medium (DMEM), Annexin V-FITC/PI kit were obtained from Yeasen Biotechnology (Shanghai) Co.Elesclomol,cy5, coumarin 6 (Cou-6) were purchased from MedChemexpress. Goat-derived anti-rabbit IgG H&L (Alexa Fluor® 488) and Goat-derived anti-mouse IgG H&L (Cy3) conjugated antibodies, Calcein-AM/PI Double Stain Kit, Mitochondrial Membrane Potential Assay Kit with JC-1, and ATP Assay Kit, all sourced from Shanghai Beyotime Biotechnology Co. The Copper (Cu) Assay Kit was purchased from the Jincheng Bioengineering Institute. Fluorescence-labeled antibodies for immune cell analysis were provided by Biolegend.

**Extraction of silk fibroin protein**

50g of tussah silk was put into 2.5L of Na_2_CO_3_ aqueous solution with a mass concentration of 0.06%, treated at a temperature of 98-100℃ for 30 minutes, repeated 4 times to degum the silk, thoroughly washed and dried to obtain pure tussah silk fibroin fiber. Pure tussah silk fibroin fiber was added to a saturated lithium bromide solution and stirred at 50 ℃ to dissolve it into a mixed solution of tussah silk fibroin protein. The obtained mixed solution of tussah silk fibroin was put into a dialysis bag and dialyzed with ddH_2_O for 4 days to remove impurities such as lithium bromide, obtaining a pure tussah silk fibroin protein solution.

**Synthesis of tussah silk fibroin nanoparticles**

The concentration of tussah silk fibroin protein solution was adjusted to 20mg/mL, CuCl_2_ with a concentration of 2mmol/mL, and the mixture was in a 40 ℃ water bath environment for 10 minutes to obtain a suspension of tussah silk fibroin protein nanoparticles. Then, the silk fibroin protein microspheres were obtained by centrifugation and freeze-drying, weighed, and the yield was 95%. According to electron microscopy observation and detection, the tussah silk fibroin protein microspheres are circular with an average diameter of 100 nanometers.

**Cell viability assays**

The CCK8 assay was utilized to assess the impact of different treatments on pancreatic cancer cell lines. Pancreatic cancer cells (Panc1, Mia, and Pan02) were seeded into 96-well plates at a density of 8000 cells per well and allowed to incubate overnight. Following this, the cells were exposed to different concentrations of the designated drugs, with ES concentrations ranging from 0.1nM to 10,000nM, for 24 hours. After treatment, the cells were further incubated with Cell Counting Kit-8 (CCK-8) reagent for 2 hours. The absorbance was then measured using a microplate reader (Tecan, Switzerland) at a wavelength of 450 nm to assess cell viability.

**Cellular uptake**

To analyze the uptake of TSF@Cou-6, we used CLSM and FCM. For CLSM observation, a cover glass was placed at the bottom of each 6-well plate. Add 1 mL of Pan02 cells (1×10^5^) into each culture medium well and incubate overnight. Subsequently, Pan02 cells were treated with TSF@Cou-6 nanoparticles for durations of 1, 2, 4, and 8 hours, respectively. The cells were subsequently rinsed three times with PBS, and their nuclei were labeled with DAPI. Subsequently, CLSM collected images. In addition, FCM was used to detect the intracellular levels of Cou-6 further. Treating the cells under the same conditions as the CLSM analysis mentioned above. Finally, the level of intracellular Cou-6 was collected and detected through FCM.

**Apoptosis assay**

Cell apoptosis was evaluated using an Annexin V-FITC apoptosis detection kit. Pan02 cells (3×10⁵ cells per well) were plated in 6-well plates and allowed to incubate overnight. On a subsequent day, the cells were exposed to various treatments: PBS, ES, Cu-TSF, ES-Cu, and TSF@ES-Cu. The final concentration of ES was set at 100nM, and the treatments were carried out for 12 hours. Following treatment, the cells were collected and incubated with Annexin V/PI staining solution in the dark at 25°C for 15 minutes. Apoptosis was then analyzed immediately by FCM.

**Cell apoptosis test in 3D tumor spheroids**

Firstly, 1% agarose gel solution (Aladdin, 50μL) was added to each 96-well plate. Then, 2000 Pan02 cells (200μL) were added to each well. On the 7th day, the cell spheres were formed. Then, the spheroids were treated with PBS, Cu-TSF, ES, ES-Cu, and TSF@ES-Cu for 24 h, respectively (The concentration of ES was 100nM). After washing with cold PBS, the 5 spheroids were stained with Calcein AM/PI Cell Viability Kit. Subsequently, images were collected with CLSM. Data were quantified by ImageJ software.

**Ros detection**

A glass slide was positioned at the base of each well in a 12-well plate, and Pan02 cells (1 × 10⁵ cells) were inoculated in 1 mL of full culture medium and incubated overnight. The following day, the cells were exposed to PBS, Cu-TSF, ES, ES-Cu, and TSF@ES-Cu for 6 hours with ES maintained at a concentration of 200nM. After the treatment, the medium was substituted with the serum-free solution. The cells were subsequently exposed to 10μM DCFH-DA, a marker for reactive oxygen species (ROS), and incubated for 20 minutes. The coverslips were mounted on microscope slides, cell nuclei were labeled with DAPI, and images were obtained using confocal laser scanning microscopy (CLSM). To further assess intracellular ROS levels, flow cytometry (FCM) was used. The cells were treated under the same conditions as for the CLSM analysis, collected, and analyzed for DCFH-DA fluorescence using FCM.

**Immunofluorescence of DLAT oligomerization and HMGB1 expression**.

Cells were subjected to the same treatment protocol as performed prior to ROS detection. Following treatment, the cells were immobilized using 4% paraformaldehyde and then incubated at 4°C overnight with an antibody targeting DLAT. The next day, the cells were exposed to a Cy5-labeled anti-mouse secondary antibody for 1 hour at room temperature, followed by a 15-minute DAPI staining at room temperature. Subsequently, images were obtained using confocal laser scanning microscopy (CLSM).

**Observation of mitochondrial morphology**

Pan02 cells were inoculated in the 10 cm dishes (1x10^6^ cells/dish) for 12 hours. Next, the cells were exposed to PBS or TSF@ES-Cu (the ES concentration of 100nM) for 24 hours. The next day, the cells were collected, fixed with electron microscopy fixative, and observed through biological TEM.

**Measurement of PDL-1 and CRT expression**

Cells were subjected to the same treatment protocol as performed prior to ROS detection. Following treatment, the cells were followed by blocking with 4% BSA at room temperature for 1 hour. They were then incubated with either αPD-L1 or CRT antibody at room temperature for 1 hour. Afterwards, the cells were exposed to Cy3 or FITC-conjugated anti-mouse secondary antibody at room temperature for 1 hour. The samples were analyzed using flow cytometry (FCM) to quantify the levels of cell surface markers. In addition, CLSM was used to further examine cellular CRT expressions. Cells were treated under the same conditions as in the FCM analysis and imaged using CLSM.

**ATP assay**

Pan02 cells were inoculated into a glass-bottomed cell culture dish (3×10^5^ cells /culture dish) overnight. Subsequently, the cells were exposed to PBS, Cu-TSF, ES, ES-Cu, or TSF@ES-Cu for 12 hours, with ES maintained at a concentration of 100nM. Subsequently, following the manufacturer's instructions, the extracellular ATP levels were quantified via luminescence using an ATP assay kit.

**BMDCs maturation in vitro**

Pan02 cells were initially exposed to different nanoparticles for 6 hours, then co-cultured with BMDCs for 24 more hours. Subsequently, the cells were rinsed with chilled PBS and collected. Mature DCs were analyzed using conjugated antibodies, including anti-CD11c FITC, anti-CD80-APC, and anti-CD86-PE.

**Establishment of Pan02 cancer modelⅠand efficacy study**

To establish a pancreatic cancer model, mouse pancreatic cancer Pan02 cells (2 × 10^6^ cells per mouse) were dispersed in PBS buffer and subcutaneously injected into C57BL/6 mice. The mice were assigned to different treatment groups randomly. They received up to four intravenous doses (on days 0, 3, 6, and 9) of the following treatments: PBS, Cu-TSF NPs, ES, ES-Cu, or TSF@ES-Cu NPs, with ES administered at a concentration of 6 mg/kg.

**In vivo biodistribution imaging**

TSF@ES-Cu was administered to tumor-bearing mice via tail vein injection. Afterwards, imaging systems were used to collect in vivo biological distribution images at 12 hours, 24 hours, 48 hours, and 72 hours, respectively. Finally, mice were euthanized 72 hours after injection for an in vitro biodistribution study. In vitro imaging of organs such as the heart, spleen, liver, kidneys, lungs, and tumors is collected and quantitatively analyzed using the IVIS spectroscopic imaging system.

**Establishment of Pan02 cancer model Ⅱ and efficacy study**

Mouse pancreatic cancer Pan02 cells (2 × 10⁶ cells per mouse) were suspended in PBS buffer and subcutaneously injected into C57BL/6 mice. The mice were then randomly assigned to different treatment groups. They received four doses of TSF@ES-Cu (on days 0, 3, 6, and 9) and αPDL-1 (on days 1, 4, 7, and 10) via intravenous injection. The treatments included: PBS, Cu-TSF NPs, ES, ES-Cu, and TSF@ES-Cu, with ES administered at a concentration of 6 mg/kg and αPDL-1 at 10 mg/kg.

**H&E staining, IHC staining, and IF staining**

On day 15 of tumor development, solid tumors were excised from tumor-bearing mice. The tissue samples were then subjected to histological analysis using H&E staining, immunohistochemistry (IHC), and immunofluorescence (IF) staining. The excised tumor and organs were fixed in a 4% paraformaldehyde solution, sliced and stained with H&E, and finally scanned the slices.

**Flow cytometric analysis**

After 15 days of treatment, tumor tissue and spleen were digested and dissociated into a single-cell suspension. Subsequently, the cells were analyzed using conjugated antibodies, including anti-CD45-BV605, anti-CD11c-FITC, anti-CD86-PE, anti-CD80-APC, anti-CD3-FITC, anti-CD4-APC, anti-CD8-FITC, anti-CD11b-FITC, anti-CDF4/80-APC and anti-CD206-PE. Finally, FCM was used to detect the cells.

**Statistical analysis**

All data were analyzed statistically using GraphPad Prism 9.0 software. Results are expressed as the mean ± standard deviation (SD). The statistical significance of the data was assessed using one-way and two-way analysis of variance (ANOVA), as well as Student’s T-test. The p-values of *p < 0.05, **p < 0.01, and ***p < 0.001 were considered statistically significant.

**2.Supplementary Figure**


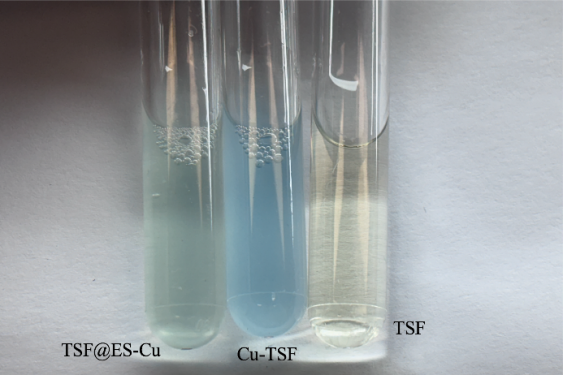


**Figure S1.** Digital photography of TSF, TSF-Cu NPs, and TSF@ES-Cu NPs.


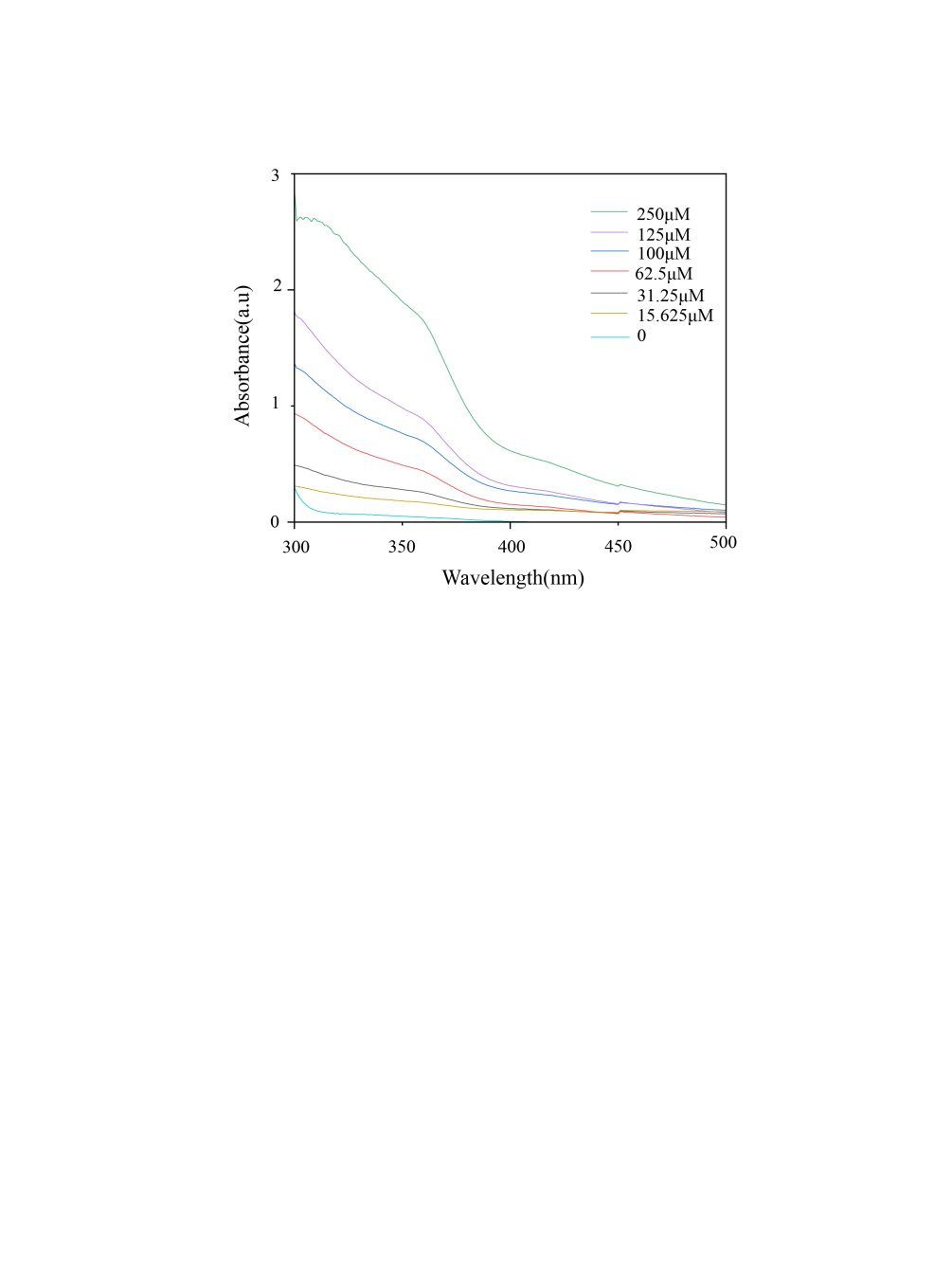


**Figure S2.** UV-vis absorption spectra of ES-Cu at different concentrations.


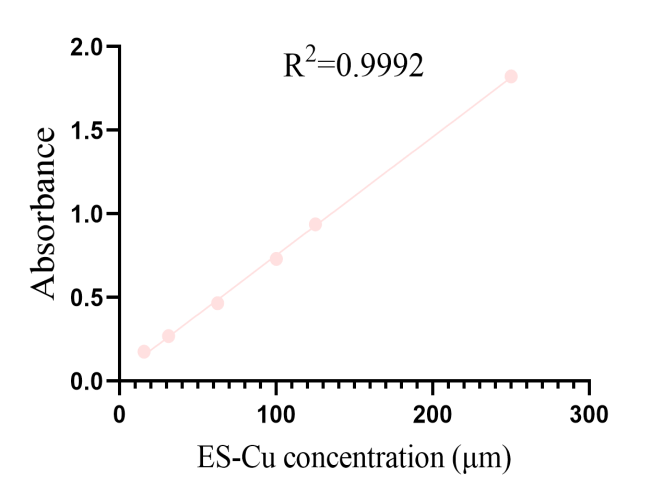


**Figure S3.** The standard curve of the absorption intensity (355nm) of ES-Cu as a function of concentration.


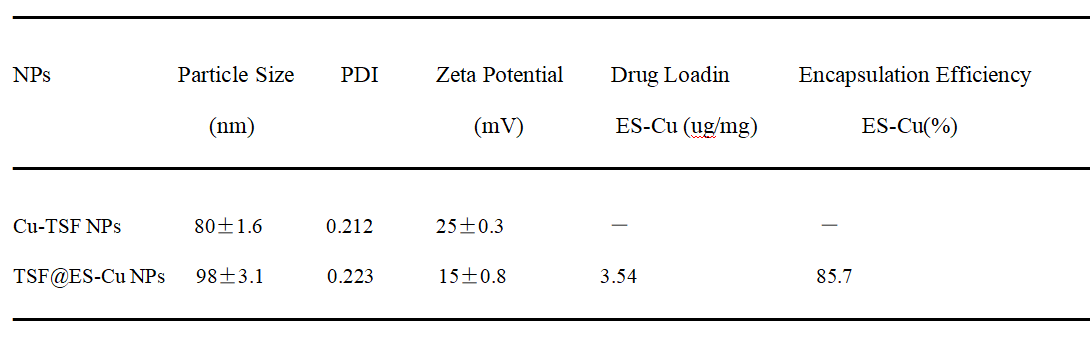


**Figure S4.** Physicochemical parameters of Cu-TSF NPs and TSF@ES-Cu NPs.


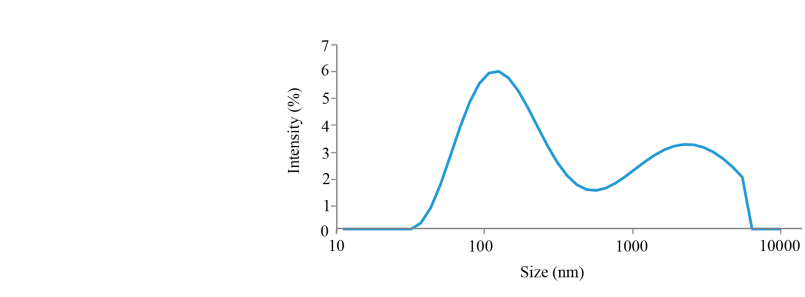


**Figure S5.** Representative particle size distribution in the presence of 1mm H_2_O_2_ for 6h.


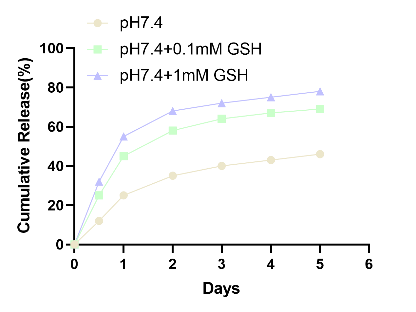

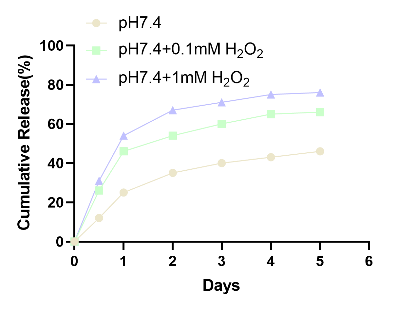

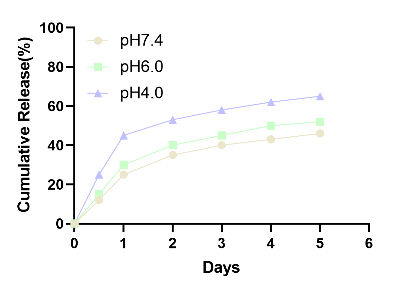


**Figure S6.** Drug-release profiles of TSF@ES-Cu NPs.

1. (b)


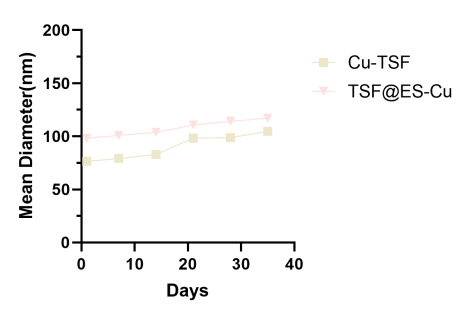

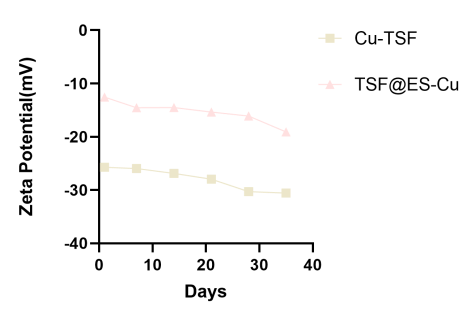


**Figure S7.** Hydrodynamic particle sizes(a) and (b) zeta-potentials of Cu-TSF NPs and TSF@ES-Cu NPs at pH 6.8 for 35 days.


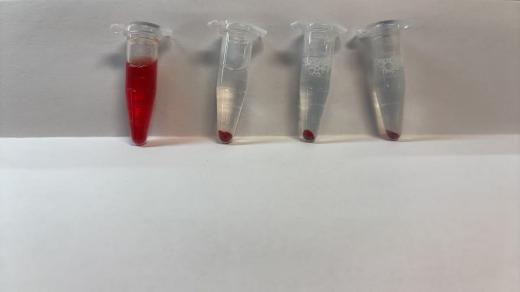


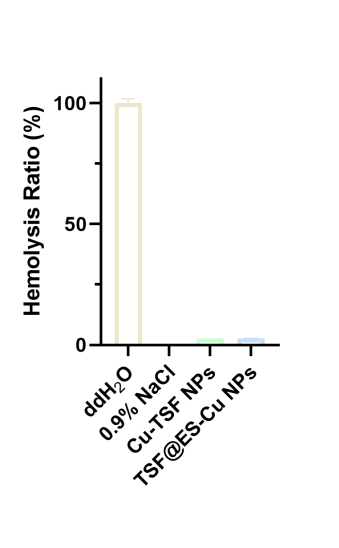


**Figure S8.** Hemolysis assay of TSF NPs and TSF@ES-Cu NPs.


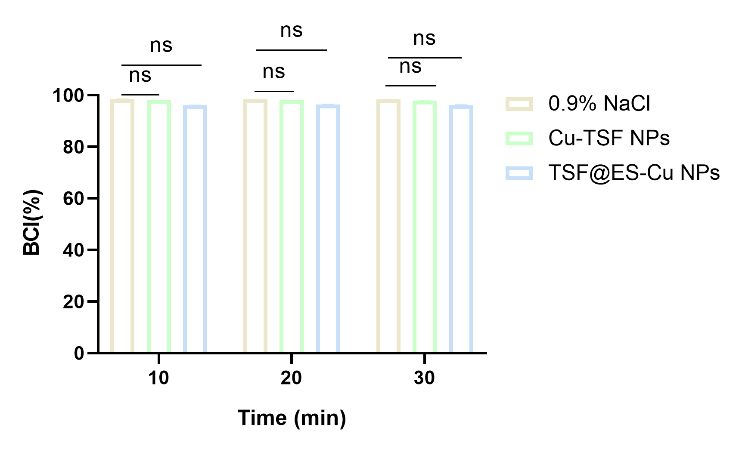


**Figure S9.** Coagulation tests TSF NPs and TSF@ES-Cu NPs.


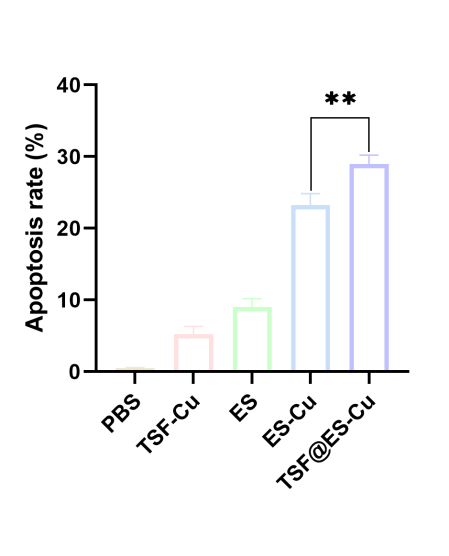


**Figure S10.** Semi-quantification of the apoptotic rate of Pan02 cells with various treatments via FCM

DAPI FITC Merge


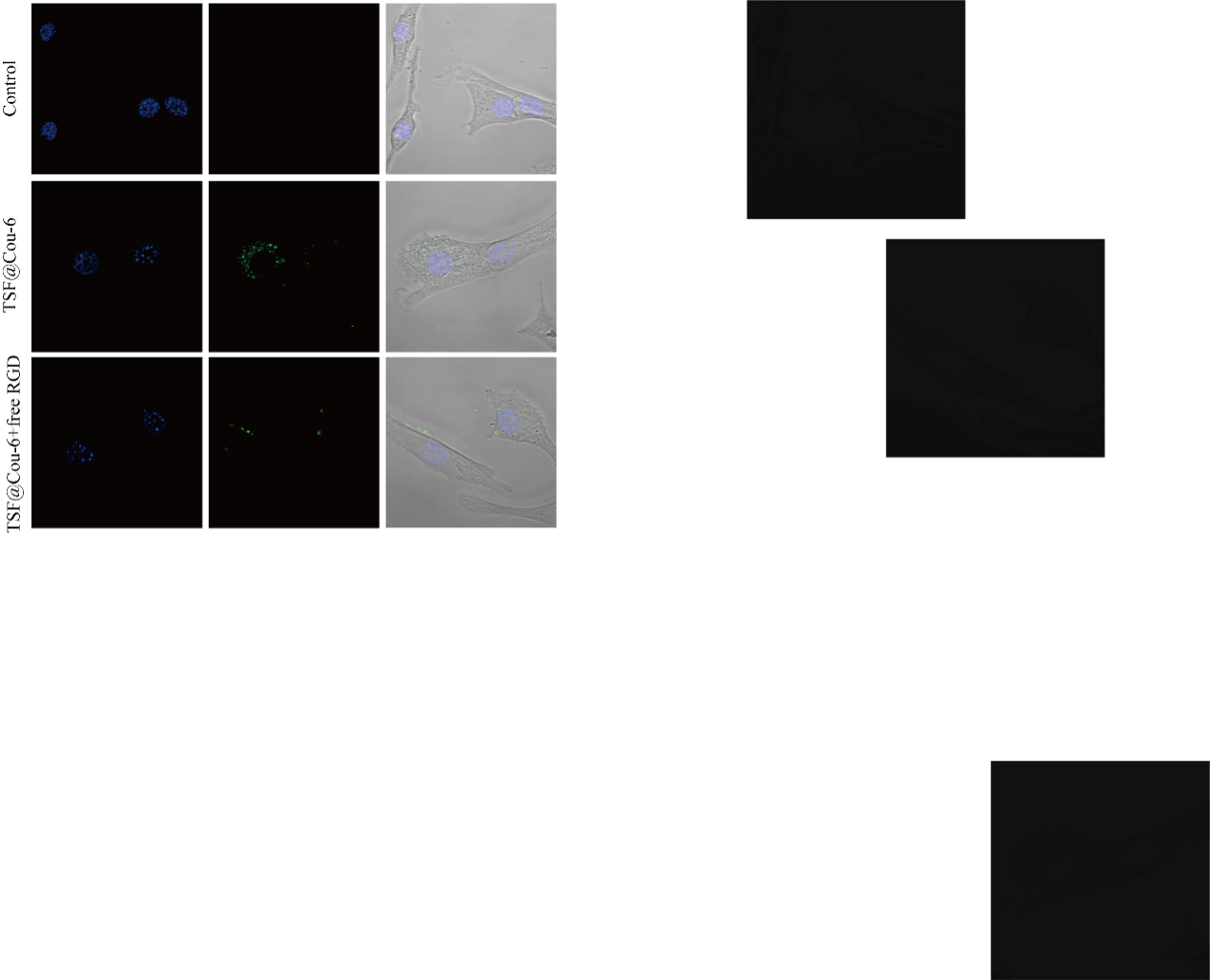


**Figure S11.** Cellular internalization of TSF@Cou-6 NPs and TSF@Cou-6 NPs in the presence of free RGD molecules by Pan02 cells after incubation for 2 h. Untreated cells were used as the negative control. Scale bar = 20 μm.


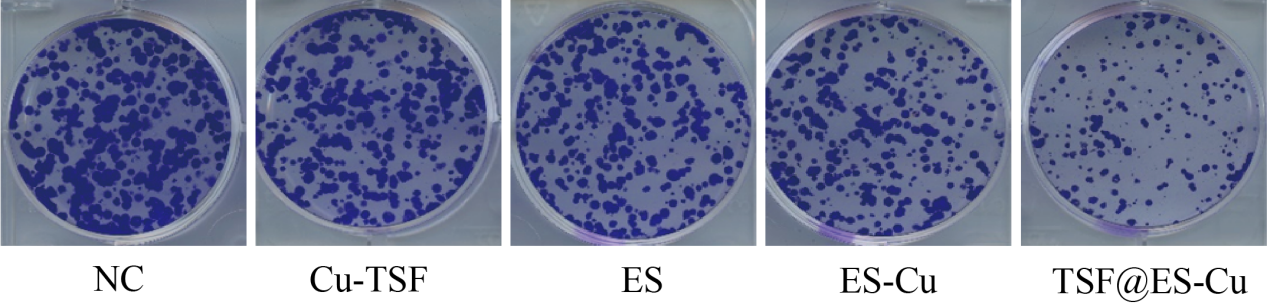


**Figure S12.**  Colony of Pan02 cells treated with different NPs.


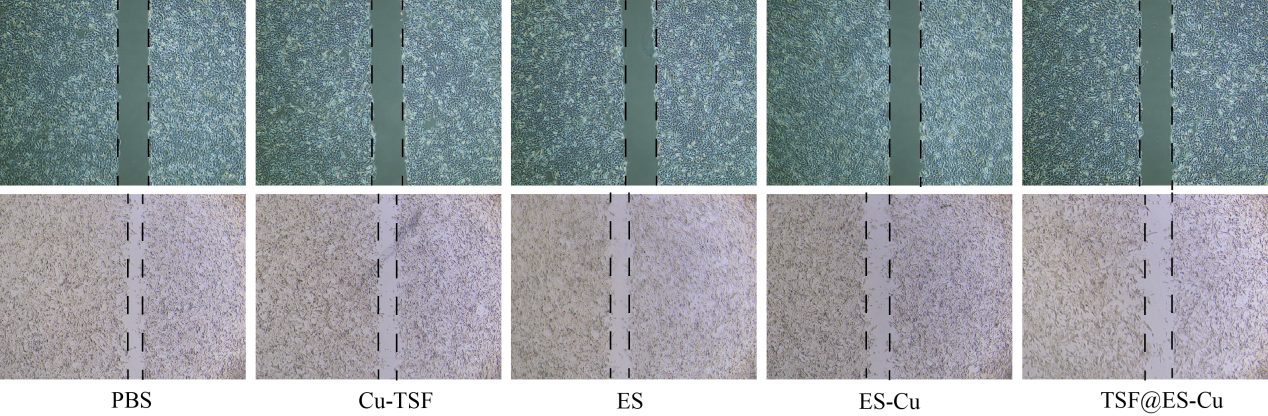


**Figure S13.**  The scratch assay of Pan02 cells treated with different NPs.


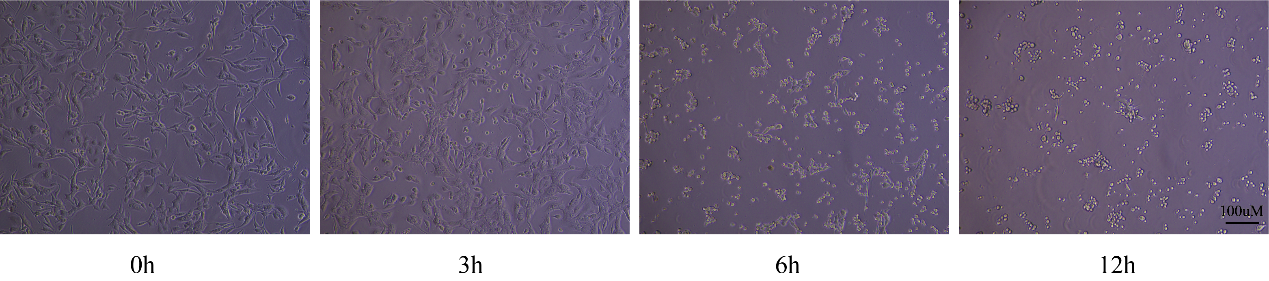


**Figure S14.** Typical morphological changes of Pan02 cells treated with TSF@ES-Cu NPs at different time periods (the concentration of ES was 200nm). Scale:100 um.


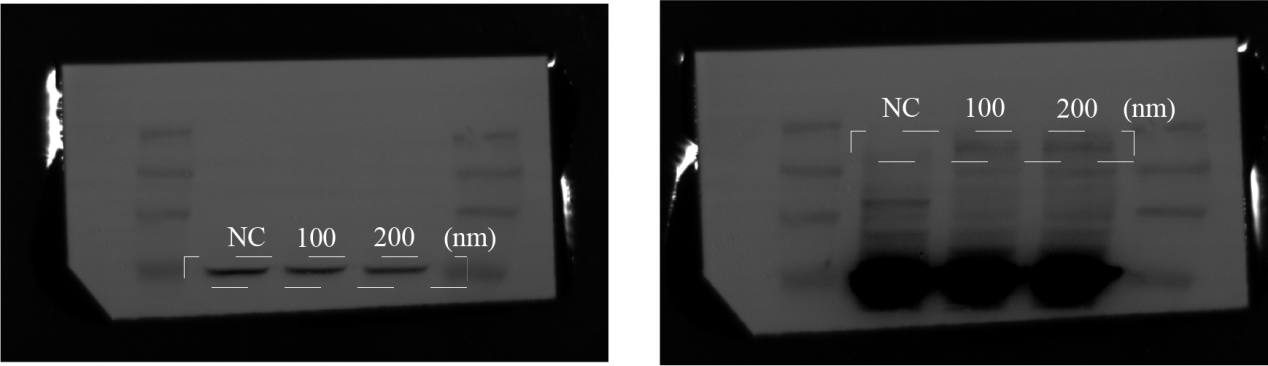


**Figure S15.** Western blot of DLAT and DLAT oligomers after treatment with different concentrations of TSF@ES-Cu NPs.


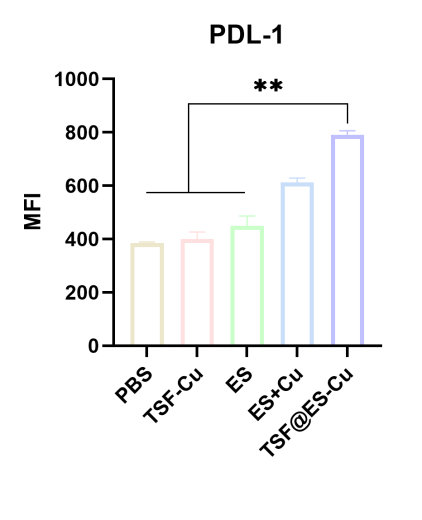


**Figure S16.** Semi-quantification of the PDL-1 expression of Pan02 cells with various treatments via FCM.


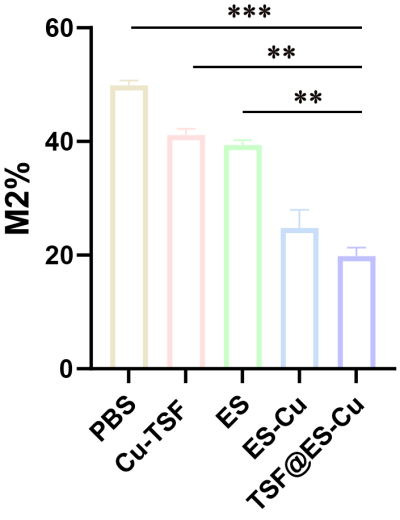


**Figure S17.** Quantitative analysis of M2 macrophage in tumors with different treatments.


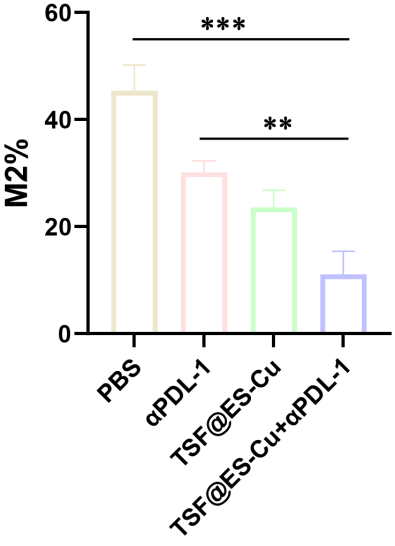


**Figure S18.** Quantitative analysis of M2 macrophage in tumors with different treatments.


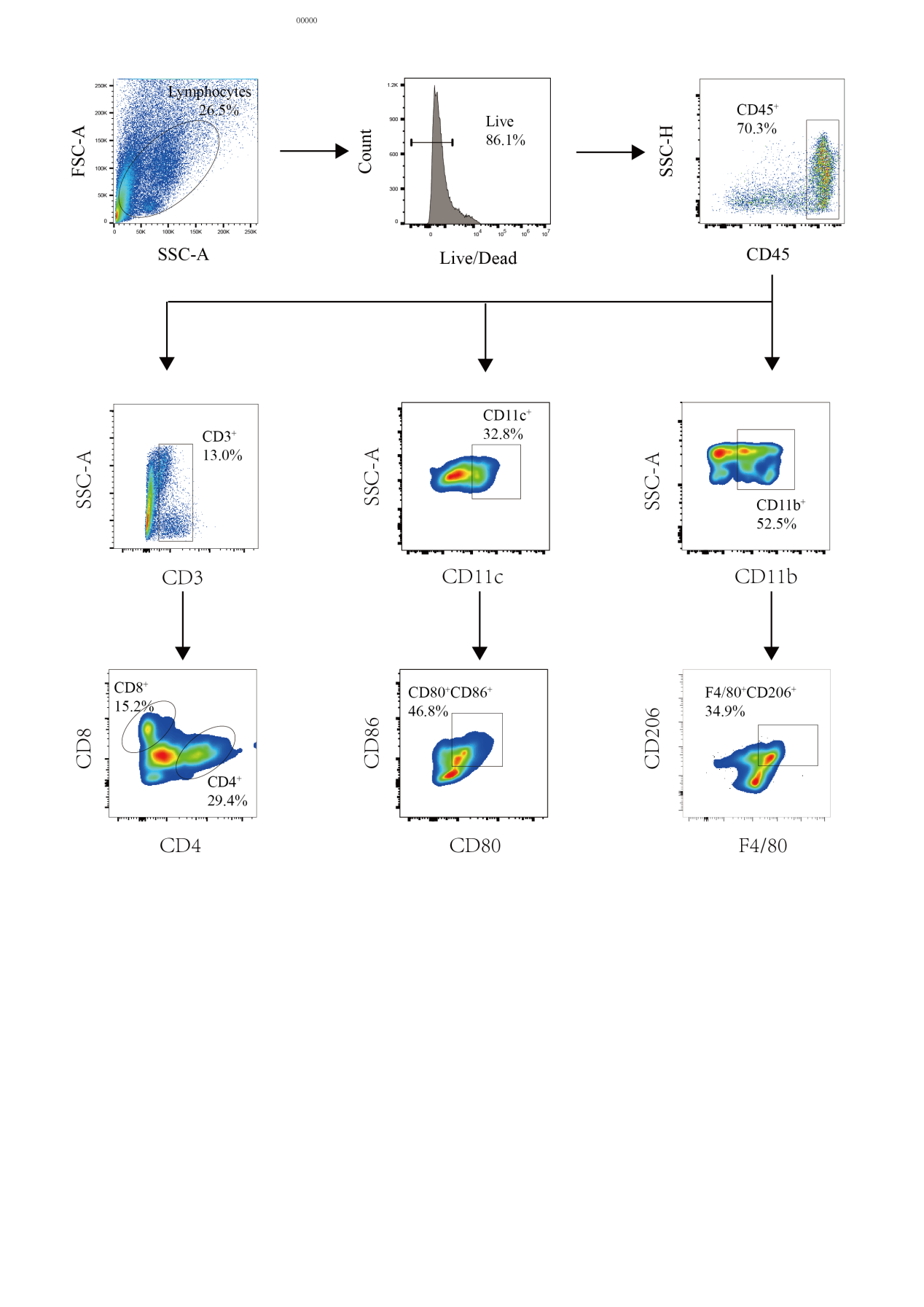


**Figure S19.** The gating strategy for tumor-infiltrating lymphocytes in tumor tissues after various treatments.
